# Dynamic Interplay between the Periplasmic and Transmembrane Domains of GspL and GspM in the Type II Secretion System

**DOI:** 10.1371/journal.pone.0079562

**Published:** 2013-11-01

**Authors:** Mathilde Lallemand, Frédéric H. Login, Natalia Guschinskaya, Camille Pineau, Géraldine Effantin, Xavier Robert, Vladimir E. Shevchik

**Affiliations:** 1 Université Lyon 1, Lyon, France; 2 INSA-Lyon, Villeurbanne, France; 3 CNRS, UMR5240, Microbiologie Adaptation et Pathogénie, Lyon, France; 4 Laboratory for Biocrystallography and Structural Biology of Therapeutic Targets, Molecular and Structural Bases of Infectious Diseases, CNRS UMR5086, Lyon, France; Centre National de la Recherche Scientifique, Aix-Marseille Université, France

## Abstract

The type II secretion system (T2SS) is a multiprotein nanomachine that transports folded proteins across the outer membrane of gram-negative bacteria. The molecular mechanisms that govern the secretion process remain poorly understood. The inner membrane components GspC, GspL and GspM possess a single transmembrane segment (TMS) and a large periplasmic region and they are thought to form a platform of unknown function. Here, using two-hybrid and pull-down assays we performed a systematic mapping of the GspC/GspL/GspM interaction regions in the plant pathogen *Dickeya dadantii*. We found that the TMS of these components interact with each other, implying a complex interaction network within the inner membrane. We also showed that the periplasmic, ferredoxin-like, domains of GspL and GspM drive homo- and heterodimerizations of these proteins. Disulfide bonding analyses revealed that the respective domain interfaces include the equivalent secondary-structure elements, suggesting alternating interactions of the periplasmic domains, L/L and M/M versus L/M. Finally, we found that displacements of the periplasmic GspM domain mediate coordinated shifts or rotations of the cognate TMS. These data suggest a plausible mechanism for signal transmission between the periplasmic and the cytoplasmic portions of the T2SS machine.

## Introduction

 The type II secretion system is a sophisticated multiprotein machine that promotes the secretion of folded proteins and protein multimers from the periplasm through the outer membrane into the external medium or host tissues. The T2SS is widespread among γ-proteobacteria and is also found in α-, β- and δ-proteobacteria [1]. However, functional and structural studies of the T2SS have mostly been performed on only a few bacterial species which are pathogenic for humans, animals, fish and plants [2,3].The T2SS of the plant pathogenic bacterium *Dickeya dadantii* (formerly *Erwinia chrysanthemi*) secretes a dozen lytic enzymes, mainly pectinases [4]. The T2SS is called the Out system in *Dickeya* and in the related *Pectobacterium* genus (formerly *Erwinia carotovora*) but it is generically called the Gsp (General secretory pathway) in any bacteria. The T2S machinery is composed of at least 12 conserved core elements, GspC to GspM and GspO, and it can be divided into three functional and structural blocks: the secretin, the multiprotein inner membrane (IM) platform, and the pilus-forming subunits. The secretin GspD forms dodecameric pore-like structures in the outer membrane through which the substrates can be translocated [5]. The N-terminal domains of GspD form a vestibule-like structure, in the periplasm, where the substrate can be docked prior to secretion [6,7]. In some bacteria, a specialized class of lipoproteins assists in the correct targeting and assembly of the secretins in the outer membrane [8–10]. The periplasmic domain of the secretin interacts with that of GspC, an inner membrane component of the T2SS [11–15]. GspC, together with the two other bitopic IM components, GspL and GspM, and a multispanning membrane protein GspF are thought to constitute a complex of unknown stoichiometry, referred to as an IM platform [16]. The ATPase GspE is attached to the inner leaflet of the IM *via* interactions with the cytoplasmic domains of GspL and GspF [16–18]. GspE can provide the energy for the machine assembly or for the secretion itself, more probably, for the formation of a short pilus by the five pseudopilins GspG to GspK [19]. It is thought that this pilus pushes the secretion substrate through the secretin pore [20,21].

 Many aspects of this hypothetical model remain elusive. Notably, it has been suggested that GspC, GspL, GspM, GspF, together with GspE, constitute an IM complex that could act as a platform for the assembly and anchoring of the pilus [16]. This platform has also been presumed to initiate signal transduction, pore gating and to provide and transmit the energy for protein secretion [2,3,16]. However, its exact function, as well as the mechanisms that govern the assembly of its components and their stoichiometry, still needs to be elucidated. GspC, GspL and GspM are bitopic IM proteins carrying a single transmembrane segment and a large periplasmic region. GspL and GspM have been found to form a stable binary complex that interacts with GspC and plays a key role in the stabilization of the IM platform [22-26]. Previous studies have shown that the periplasmic regions of GspL and GspM self-interact and interact with each other, while the cytoplasmic domain of GspL self-dimerizes and interacts with GspE [23,27-32]. Even though the exact position of the corresponding interaction sites is still uncertain depending on the bacteria and the approaches used, it is generally believed that the TMSs are not necessary for the interactions of these IM components. However, recent studies show that the full functions of GspC and GspM require the self-interaction of their respective TMSs [33,34]. Therefore, the relevance of the TMSs in the assembly and function of the IM platform needs to be reconsidered. 

 Recent structural studies have shed new light on the assembly of the IM platform. Notably, they have revealed that the periplasmic domains of GspL and GspM adopt a similar ferredoxin-like (FL) fold [29,32]. These studies have also highlighted the common evolutionary origin of the T2SS and the type IV pili (T4P) and have shown that all the IM core components of the T2SS have a structural ortholog within the T4P. More precisely, the GspC/L/M components adopt folds which are similar to those of PilP/M/N/O, where PilM and PilN correspond to the cytoplasmic and periplasmic regions of GspL, respectively [29,35-39]. Although the periplasmic regions of GspL and GspM, as well as PilN and PilO, adopt a similar FL fold, the organization of the subunits in the corresponding crystallographic dimers appears to be completely different [29,32,36]. This suggests that there are some essential differences in the assembly and function of these components within the respective systems. Alternatively, the biological relevance of the assumed inter-domain interfaces may be in question and needs to be examined further.

 Here, using pull-down and two-hybrid assays we performed a systematic mapping of the GspC/GspL/GspM interaction regions of the T2SS in the plant pathogenic bacteria *D. dadantii*, respectively OutC/OutL/OutM. We found that the transmembrane segments of these components interact with one another, implying a complex interactive network within the IM. Two-hybrid assays showed that homo- and heterodimerizations of the FL domains (FLD) of OutL and OutM are rather exclusive, implying an overlap of the respective interaction sites. At the same time, disulfide bonding analyses indicated that the FLD/FLD interfaces formed by OutL and OutM include the equivalent secondary-structure elements, suggesting alternating FLD interactions, L/L and M/M versus L/M. Disulfide-bonding analysis, applied to the transmembrane segment of OutM, indicates that these displacements of the FL domains induce coordinated movements or rotations of the cognate TMSs.

## Results

### Interactions between the full-length OutC, OutL and OutM

**Figure 1:**
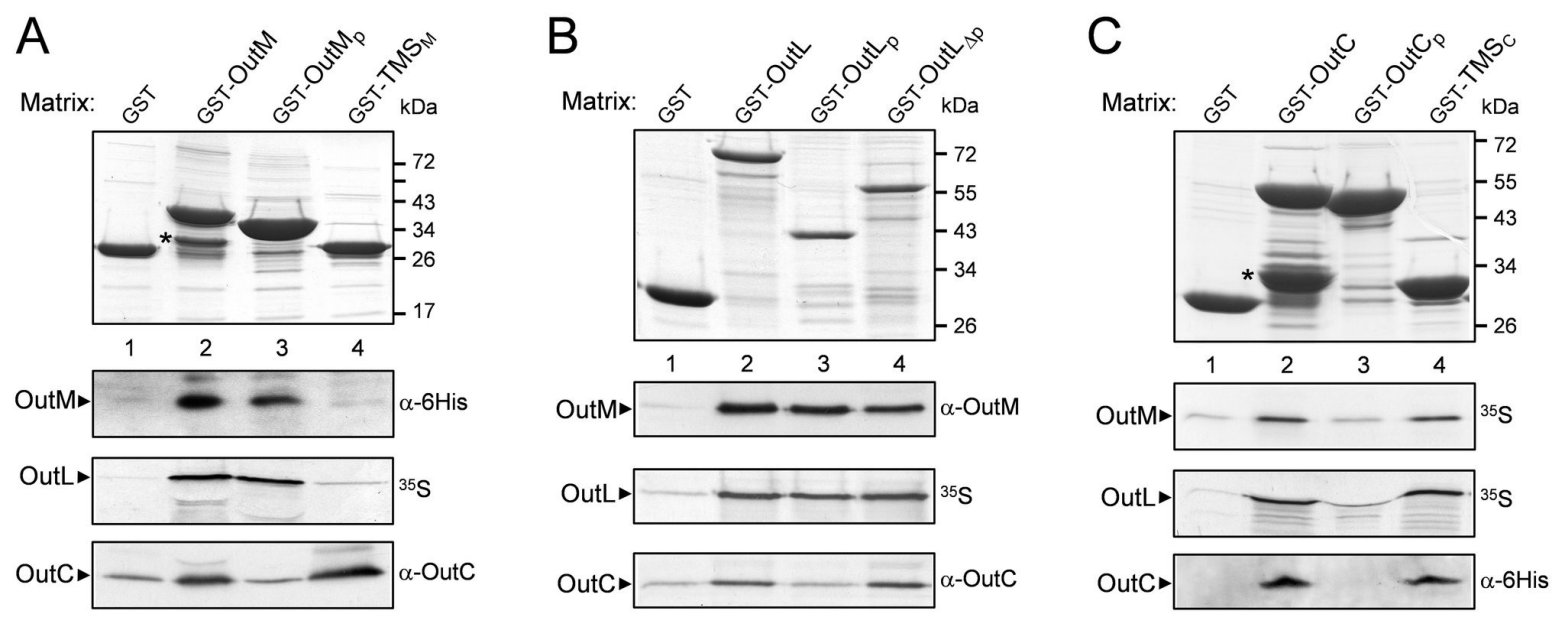
Dissection of the interacting regions of OutC, OutL and OutM in pull-down assays. The GST-fused derivatives of OutM (*A*), OutL (*B*) or OutC (*C*) (indicated at the top) were immobilized on Glutathione Sepharose beads, to constitute the affinity matrices (upper panels). Next, the indicated proteins of interest were incubated with these matrices for 1 h and unbound proteins were washed away. Bound proteins were eluted with Laemmli sample buffer, separated by SDS-PAGE and either stained (upper panels), or (lower panels) probed with the indicated antibodies, or revealed by autoradiography (^35^S). GST-fused degradation products are indicated by asterisks. Schematic representation of the used derivatives is shown in Figure S1.

 First, a GST pull-down assay was used to search for potential bi-partner interactions between OutC, OutL and OutM. The full-length proteins, carrying a GST-tag, were bound onto Glutathione Sepharose and used as baits, whereas those carrying a His-tag were solubilized with Triton X-100 and used as prey in the liquid phase. These assays revealed that each of the three proteins interacts *in vitro* with itself and with the two other proteins (Figure 1A-C, compare lanes 1 and 2). 

**Figure 2:**
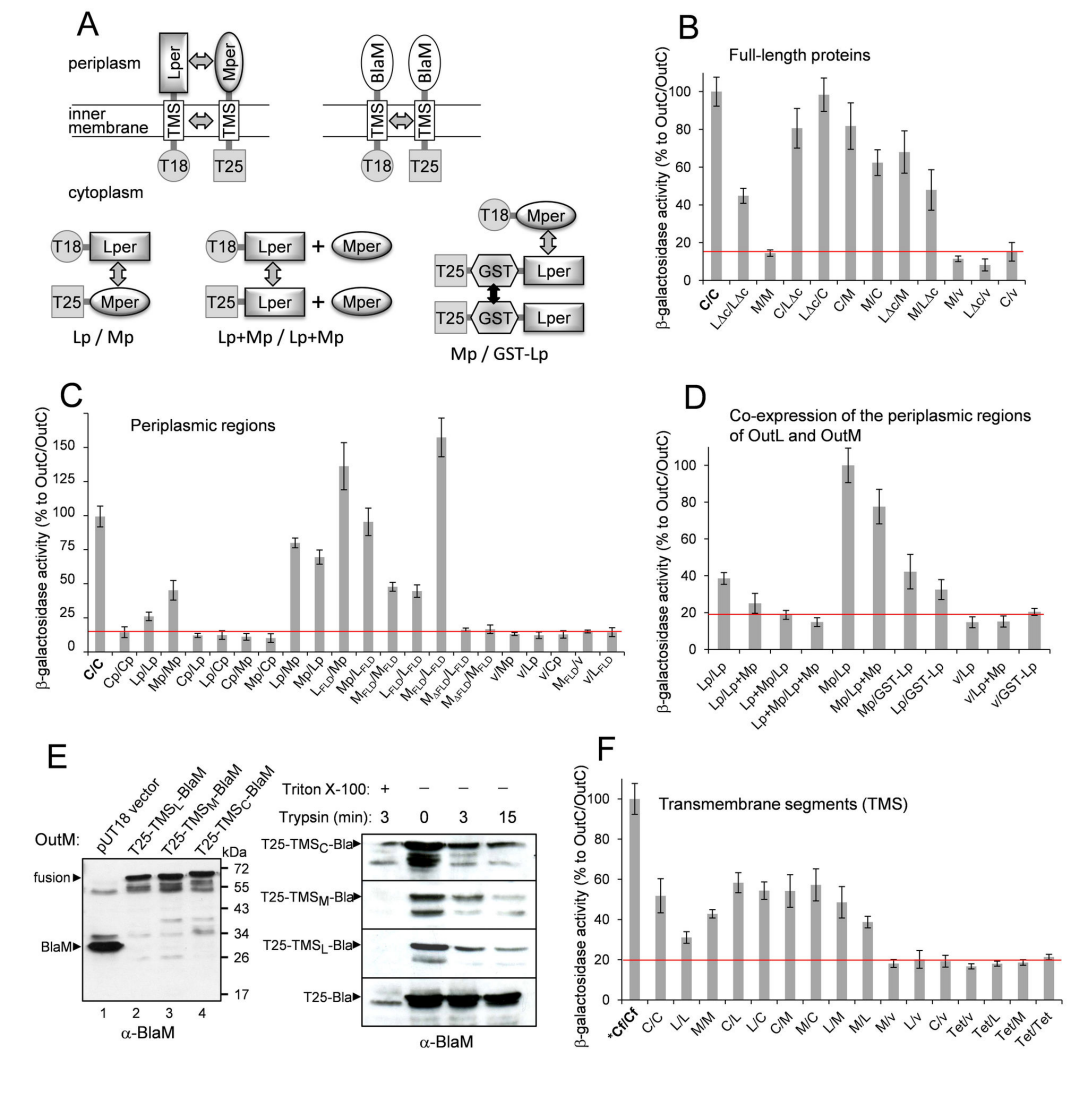
Bacterial two-hybrid assays map the interacting regions of OutC, OutL and OutM. (*A*), the fusions of T18(T25) to either the full-length proteins (top left), or the transmembrane segments (top right), or the periplasmic regions (bottom) were generated and used in two-hybrid assays as shown here. The fusions of the full-length proteins (*B*), the periplasmic regions (*C* and *D*) or the transmembrane segments (*F*) of OutC, OutL and OutM were co-expressed in *E. coli* DHP1 *cyaA* strain. The values of β-galactosidase observed with each pair (T18-fusion/T25-fusion) are expressed as percentage of that with the full-length OutC/OutC, considered as 100% and indicated as C/C (*B* and *C*) or *Cf/Cf (*F*). In (*F*), the triple T18(T25)-TMS-BlaM fusions are denoted as C, L and M, for an easy reading. All assays were performed from triplicate culture on three to four different transformants, standard deviations are indicated. (*E*), to check the integrity of the triple T25-TMS-BlaM fusions, *E. coli* cells expressing these fusions (indicated at the top) were probed by immunoblotting with BlaM-antibodies (left panel). To assess the correct insertion of these fusions into the inner membrane, as shown in (*A*), *E. coli* cells expressing the fusions were converted into spheroplasts and treated with trypsin for the indicated time periods (right panel). In contrast to the T25-BlaM fusion that remained in the cytoplasm, the triple T25-TMS-BlaM fusions were exposed in the periplasm since they were degraded by trypsin.

 Next, the bacterial two-hybrid system [40] was employed to investigate potential interactions between OutC, OutL and OutM *in vivo*. This technique was initially developed to assess the interactions between soluble proteins in the *E. coli* cytoplasm but it is also compatible with bitopic IM proteins [33,41,42]. In the last case, the T18 or T25 domain of adenylate cyclase, CyaA, is fused to the N-terminus of a full-length membrane protein and, consequently it remains in the cytoplasm (Figure 2A, top left panel). As a result, protein-protein interactions that occur in the IM and/or in the periplasm direct the reconstitution of CyaA activity in the cytoplasm and, hence, the expression of the reporter genes (*via* cAMP synthesis). Here, fusions of T18 or T25 to the full-length OutC and OutM were employed. Since the fusions with the full-length OutL were not stable (data not shown), OutL∆cyt, lacking the cytoplasmic region, was used instead (Figure S1A). The tested protein combinations, except for OutM/OutM pair, produced significant levels of β-galactosidase, indicating that the three proteins interact with each other; in addition, OutC and OutL∆cyt self-interact (Figure 2B). With the exception of the OutM/OutM pair, these data are fully consistent with the results of the pull-down assays and they indicate that the periplasmic regions and/or the TMSs of OutC, OutL and OutM may interact.

### Dissection of the periplasmic interactions

 To test this hypothesis, the periplasmic regions of the three proteins (Figure S1A) were probed in a pull-down assay. OutM and OutL interacted with both GST-OutMp and GST-OutLp, whereas OutC did not (Figure 1A and B, compare lanes 1 and 3). In addition, no protein was bound to GST-OutCp (Figure 1C, lane 3), indicating that the periplasmic region of OutC does not interact with itself or with that of OutM and OutL. The two-hybrid assays showed similar results: the levels of β-galactosidase indicated a strong interaction between OutLp and OutMp and a rather weak homo-dimerization for each of them (Figure 2C). No interaction involving the periplasmic region of OutC was detected.

**Figure 3:**
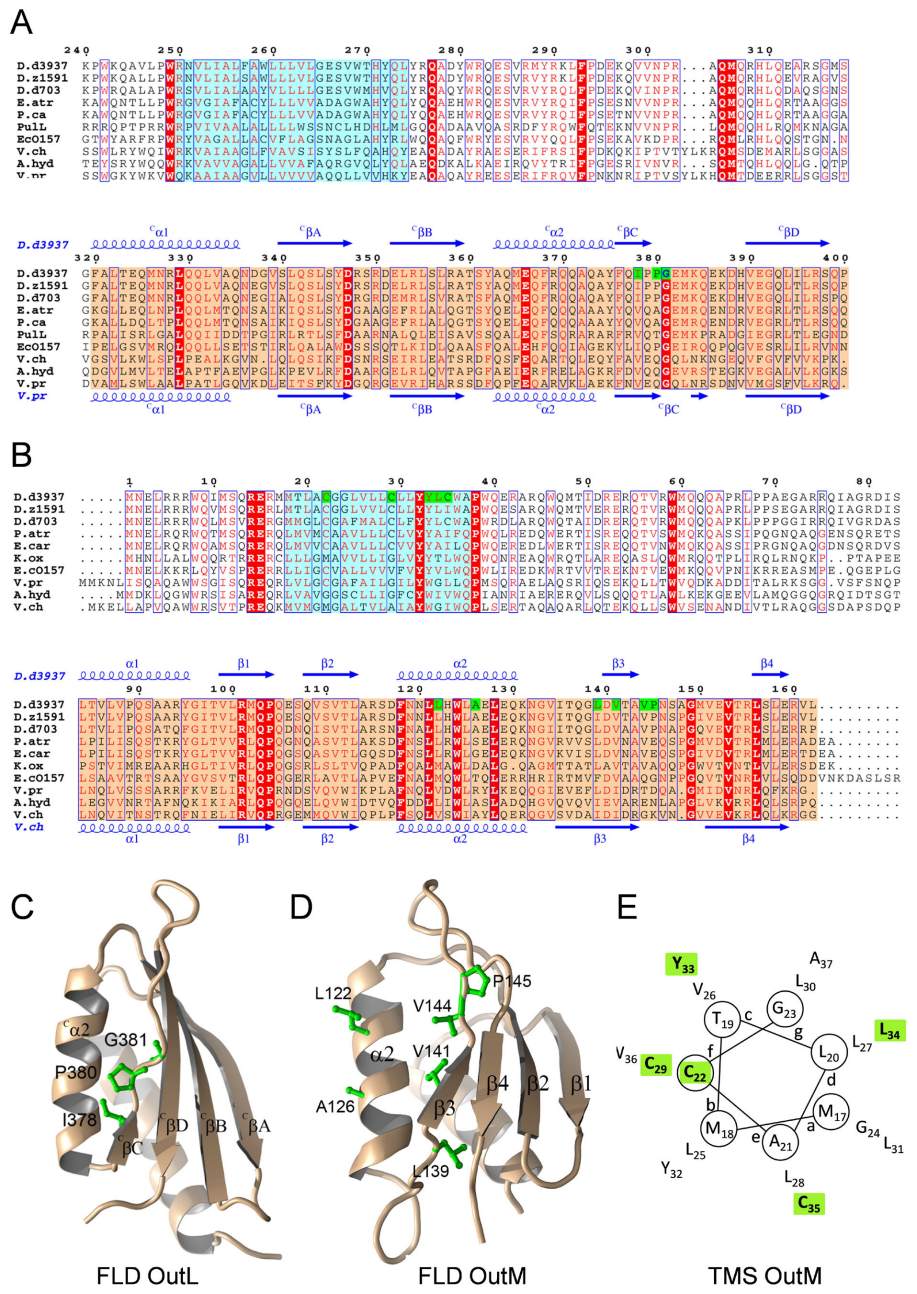
Overview of the positions in OutL and OutM selected for mutagenesis. (*A* and *B*), multiple sequence alignments of representative examples of OutL (*A*) and OutM (*B*) homologues (UniProt/NCBI accession codes are listed in Supplemental section). The residue numbering is for *D. dadantii* 3937 OutL and OutM, respectively. The secondary structure elements corresponding to the crystal structures of the periplasmic domain of EpsL from *V. parahaemolyticus* (PDB entry 2W7V) and of EpsM from *V. cholerae* (PDB entry 1UV7) are indicated at the bottom and those of the modeled OutL and OutM at the top, respectively. The equivalent secondary elements of OutL and OutM possess different denominations, according to the original reports [29,32]. Residues in red or in red background are similar and identical, respectively. The positions of the TM segment (determined with TMHMM Server v2.0 [59]) and the FL domain are indicated in cyan and orange backgrounds, respectively. The residues mutated in this study are highlighted in green. Alignment was generated using the ENDscript/ESPript web server [60,61]. (*C* and *D*), the models of OutL (residues 311 to 400) and OutM (residues 83 to 162) were generated with the homology molecular modeling program MODELLER 9v11 [57] by using as templates the structure of the periplasmic domain of EpsL from *V. parahaemolyticus* and of EpsM from *V. cholerae*. The residues mutated in this study are represented as green sticks. Figure was produced using PyMOL [62]. (*E*), the helical wheel projection of the predicted TM α-helix of OutM. The residues mutated in this study are highlighted in green.

 Structural studies have revealed that the C-terminal regions of EpsL from *V. parahaemolyticus* and EpsM from *V. cholerae* (23 % and 28 % of identity with the equivalent regions of OutL and OutM, respectively) adopt a similar ferredoxin-like (FL) fold (Figure 3) [29,32]. When the FL domains of OutL and OutM, denoted L_FLD_ and M_FLD_ respectively, were assessed in a two-hybrid assay, the levels of β-galactosidase were even higher than those observed with the entire periplasmic regions (Figure 2C, compare Lp/Mp with L_FLD_/Mp and Mp/Lp with M_FLD_/L_FLD_). This indicates that the FL domains drive the dimerization of the respective periplasmic regions. Consistent with this, OutM_98-162_ (M_ΔFLD_), lacking a short N-terminal section of the FLD, did not interact with OutL_FLD_ and OutM_FLD_ (Figure 2C), indicating that integrity of the domain is essential for these interactions.

### Pull-down assays suggest multiple interactions between the TMSs of OutC, OutL and OutM

The present data from the two-hybrid and pull-down assays do not explain how the full-length OutC interacts with OutL and OutM. It cannot be excluded that removal of the N-terminal part of OutC affects the remaining periplasmic region and, consequently, prevents its interactions. Alternatively, the TMSs of these proteins may interact. To investigate this possibility, the TMSs of OutC and OutM (TMS_C_ and TMS_M_, respectively) were probed in a GST pull-down assay (Figures 1 and S1A). Since GST-TMS_L_ was not stable (data not shown), GST-OutLΔp, carrying the TMS together with the cytoplasmic region, was used as an alternative. These assays showed that the full-length OutL, OutM and OutC bind to GST-TMS_C_ (Figure 1C, lane 4). Reciprocally, the full-length OutC bind to GST-TMS_M_ and to GST-OutLΔp (Figure 1A and B, lane 4). These data indicate that the TMS of OutC interacts with itself and with the TMSs of OutL and OutM. On the other hand, an interaction between the TMS of OutL and OutM remains uncertain. Indeed, neither OutL nor OutM bind to GST-TMS_M_ (Figure 1A, lane 4), suggesting that the TMS of OutM does not interact with itself or with the TMS of OutL. However, tested in the other direction, OutM does bind to OutLΔp (Figure 1B, lane 4), suggesting an interaction between TMS_L_ and TMS_M_.

### Adaptation of the two-hybrid assay for TMS-TMS interactions

 To investigate further these putative TMS-TMS interactions *in vivo*, we adapted the two-hybrid assay. The N-terminus of the TMS of interest was fused to either T18 or T25 domain of CyaA, while the C-terminus of TMS was fused to β-lactamase (Figure 2A, top right panel). Since BlaM is a monomeric protein of a size comparable with that of the periplasmic regions of OutC, OutM and OutL, its fusion to a TMS would be compatible with the studied TMS-TMS interactions. Moreover, β-lactamase allows to check the correct topology of the TMS in the inner membrane. A correct (N-in/C-out) insertion of the triple fusion directs the BlaM moiety into the periplasm and, hence, provides resistance to ampicillin. For this reason, the *blaM* gene of the pUT18C vector was replaced by *cat* (Cm^R^). All the generated triple fusions (T25-TMS-BlaM) were found to be ampicillin-resistant (up to 200 µg.ml^-1^) and trypsin-sensitive in a spheroplast assay (Figure 2E) demonstrating the periplasmic location of BlaM. Conversely, a T25-BlaM fusion, lacking any TMS, was ampicillin-sensitive and trypsin-resistant.

 The pairwise combinations of TMS_C_, TMS_L_ and TMS_M_ generated variable, although noticeable, levels of β-galactosidase indicating multiple bipartner interactions between these TMSs (Figure 2F). Consistent with the pull-down data, the two-hybrid assays showed that TMS_C_ interacts with itself and also with TMS_L_ and TMS_M_. In addition, the TMS_L_-TMS_M_ interaction, ambiguous in the pull-down assay, was clearly detected in the two-hybrid assay (Figure 2F). Moreover, two-hybrid analysis revealed a TMS_M_-TMS_M_ interaction, which was not seen in the pull-down results.

 The latter discrepancy suggests that certain conditions (e.g. the detergent used in the pull-down assay) could impair some interactions. Alternatively, the two-hybrid assay may provoke some false interactions. To test this, an “inert” TMS, the first TMS of the tetracycline resistance protein TetA, was probed as a control and fused between T18 and BlaM. When the TetA fusion was combined with a fusion carrying either TMS_L_ or TMS_M_, only a basal level of β-galactosidase was observed indicating an absence of any false positive interactions (Figure 2F).

### Competition between homo- and heterodimerization of the periplasmic regions of OutL and OutM

 The above data demonstrate multiple binary interactions between OutL, OutM and OutC, implicating their TMSs and periplasmic domains. Notably, the periplasmic domains of OutL and OutM are capable of both homo- and heterodimerizations. To test whether these interactions are synergistic or competing, we examined the effect of OutMp on the OutLp/OutLp interaction. Precisely, the isolated OutMp derivative was co-expressed with the T18-OutLp fusion from the same pUT18C vector (Figure 2A, bottom middle panel). A similar protein combination, T25-Lp + Mp, was generated on a pKT25 plasmid. Co-expression of OutMp with the T18-OutLp/T25-OutLp pair, decreased the β-galactosidase level (Figure 2D, compare the four left columns) suggesting that OutMp impairs the homodimerization of OutLp. To investigate, whether reciprocally, the homodimerization of OutLp affects its interaction with OutMp, we took advantage of the intrinsic dimerization of Glutathione S-transferase. To force homodimerization of OutLp, GST was fused between T25 and OutLp resulting in the T25-GST-OutLp triple fusion (Figure 2A bottom right panel). The insertion of GST did not affect the homodimerization of OutLp (Figure 2D, compare Lp/Lp with Lp/GST-Lp) but it decreased the interaction between OutLp and OutMp (Figure 2D, compare Mp/Lp with Mp/GST-Lp). Even if, it cannot be excluded that GST itself can impede the Lp/Mp interaction, the latter data suggest that an artificial dimerization of OutLp impairs its interaction with OutMp. Together, the coexpression experiments (Figure 2D) suggest that the homo- and heterodimerizations of the periplasmic domains of OutL and OutM are competing and this can imply an overlap of the respective interaction sites. To address further this question, we performed *in vivo* disulfide-bonding analysis.

### Cysteine mutagenesis of the FL domain of OutM

 The latter hypothesis is consistent with the structural analysis suggesting that the PilO/PilO and PilN/PilO dimers (the T4P orthologs of OutM/OutM and OutLp/OutM, respectively) may have a similar overall organization with the equivalent secondary structure elements in the interface [36]. To investigate this hypothesis, we first probed the presumed OutM_FLD_ dimer interface using cysteine mutagenesis and a disulfide-bonding analysis. To estimate the proximity of considered positions, we assessed the spontaneous formation of disulfide bonds during growth of the culture. The oxidative environment in the periplasm allows for the formation of disulfide bonds to an extent that correlates with the distance and correct orientation of the cysteine side chains [43].

**Figure 4:**
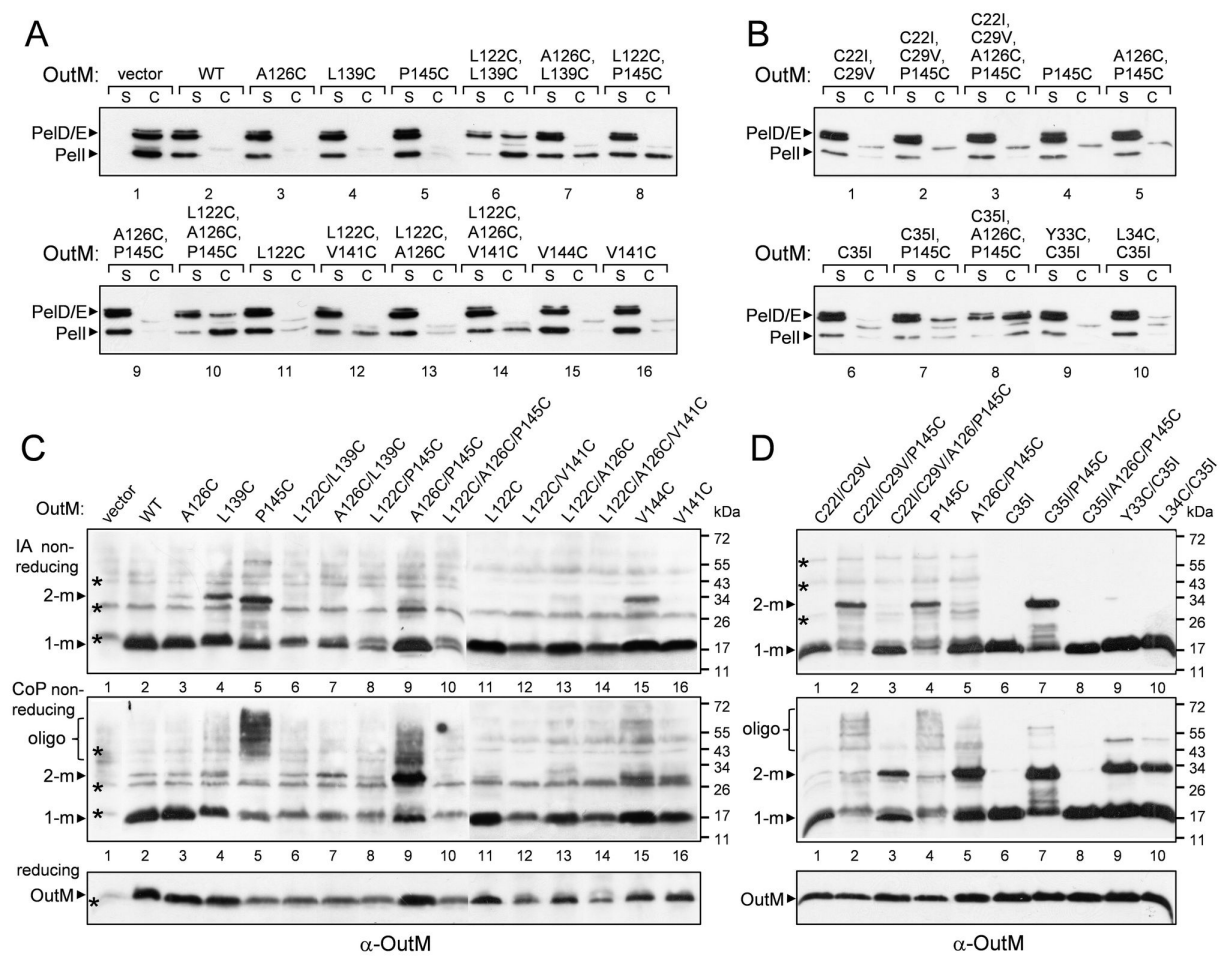
Functionality and disulfide-bonding patterns of cysteine variants of OutM. (*A* and *B*), secretion activity of OutM variants. *D. dadantii* A5269 *outM* cells, carrying a pTdB-oM plasmid with mutant *outM* alleles (indicated on top), were grown aerobically to steady-state and then the culture supernatant (s) and cells (c) were separated and analyzed by immunoblotting with PelD and PelI-antibodies. The quantity of secreted proteins present in the culture supernatant reflects the efficiency of secretion. (*C* and *D*), disulfide-bonding analysis of OutM variants. Cells from the same cultures as in (*A*) and (*B*) were either directly treated with iodoacetamide, to block any remaining free thiol groups (upper panels) or were firstly incubated with the oxidation catalyst CoPh, before the iodoacetamide treatment (middle panels). Then, the extent of disulfide bonding was assessed using non-reducing SDS-PAGE, followed by immunoblotting with OutM-antibodies. To estimate the quantity of each OutM variant, the same samples were analyzed in reducing conditions with 2-mercaptoethanol (lower panels). An equivalent amount of cells was loaded into each well. The positions of OutM monomers (1-m), dimers (2-m) and oligomers (oligo) are shown by arrowheads and non-specific specie interacting with OutM-antibodies are indicated by asterisks. The relative amount of homodimer formed by each variant reflects the proximity of the respective residue to the same residue of an adjacent OutM protomer.

 The structural study of EpsM, OutM homolog from *V. cholerae* has indicated that the FLD dimer interface involves mainly residues of the α2 helix [29]. A somewhat different FLD interface, including both the α2 helix and β3strand, has been suggested for PilO [36]. Consequently, selected OutM residues located at or close to the presumed FLD interface, namely L122 and A126 (α2 helix) together with L139 and V141 (β3 strand) and two contiguous residues V144 and P145, were substituted with cysteine (Figure 3B and D). All the single variants fully restored pectinase secretion in the *D. dadantii outM* strain (Figure 4A). Non-reducing gel showed that the variants L122C, A126C and V141C remained monomeric while L139C, V144C and, particularly, P145C generated significant quantities of homodimers (Figure 4C, upper panel, lanes 4, 5 and 15). This indicates that the latter residues in adjacent OutM protomers are proximal and, thus, the OutM_FLD_ interface includes the β3 strand. To examine further the possible α2-α2 and α2-β3 contacts suggested by the crystal structures of EpsM and PilO dimers, the substitutions in α2 and β3 were combined pairwise. The double variants, with the exception of L122C/A126C and A126C/P145C, were less abundant (Figure 4C, lower panel) but pectinase secretion was only severely impaired with L122C/L139C (Figure 4A, lane 6). Unexpectedly, none of the double variants generated a detectable quantity of homodimers (Figure 4C upper panel, lanes 6-9, 12 and 13). Moreover, L139C and P145C substitutions no longer generated dimers when a second substitution was introduced in the α2 helix (L122C or A126C). This suggests that substitutions in the α2 helix alter the arrangement of the β3 strands in the dimer interface. Supporting this idea, a combination of certain substitutions in α2 and β3 specifically interfered with the protein function. Indeed, the OutM^L122C/L139C^ variant was secretion-deficient, even though it was produced at a similar level as the functional variants OutM^L122C/V141C^ and OutM^A126C/L139C^. Since the combination of even three “permissive” cysteine substitutions, namely L122C/A126C/V141C, did not impair secretion, multiple cysteine substitutions *per se* are compatible with the protein function (Figure 4A and C, lane 14). Together, these data show that the OutM_FLD_ interface includes the residues of the β3 strand and the adjacent stretch (L139, V144 and P145) while the residues of the α2 helix (L122 and A126) are probably not directly involved in the interface but are important for the proper arrangement of the β3 strand. 

### Cysteine mutagenesis of the ferredoxin-like domain of OutL

 Using the same approach, we examined whether the ^c^βC strand of OutL, equivalent to the β3 strand in OutM (Figure 3), may also be involved in the FLD interfaces of OutL/L and OutL/M dimers. Consequently, the OutL variants I378C, P380C and G381C (^c^βC) (Figure 3A and C) were co-expressed from the same plasmid with the wild-type OutM in the *D. dadantii* ∆*outL* A5434 strain (when expressed alone in this background, OutL provoked significant growth defects). The three OutL variants were produced at the wild type level and generated comparable quantities of homodimers (Figure S2A). This suggests that the ^c^βC strand is involved in the OutL/L FLD interface.

**Figure 5:**
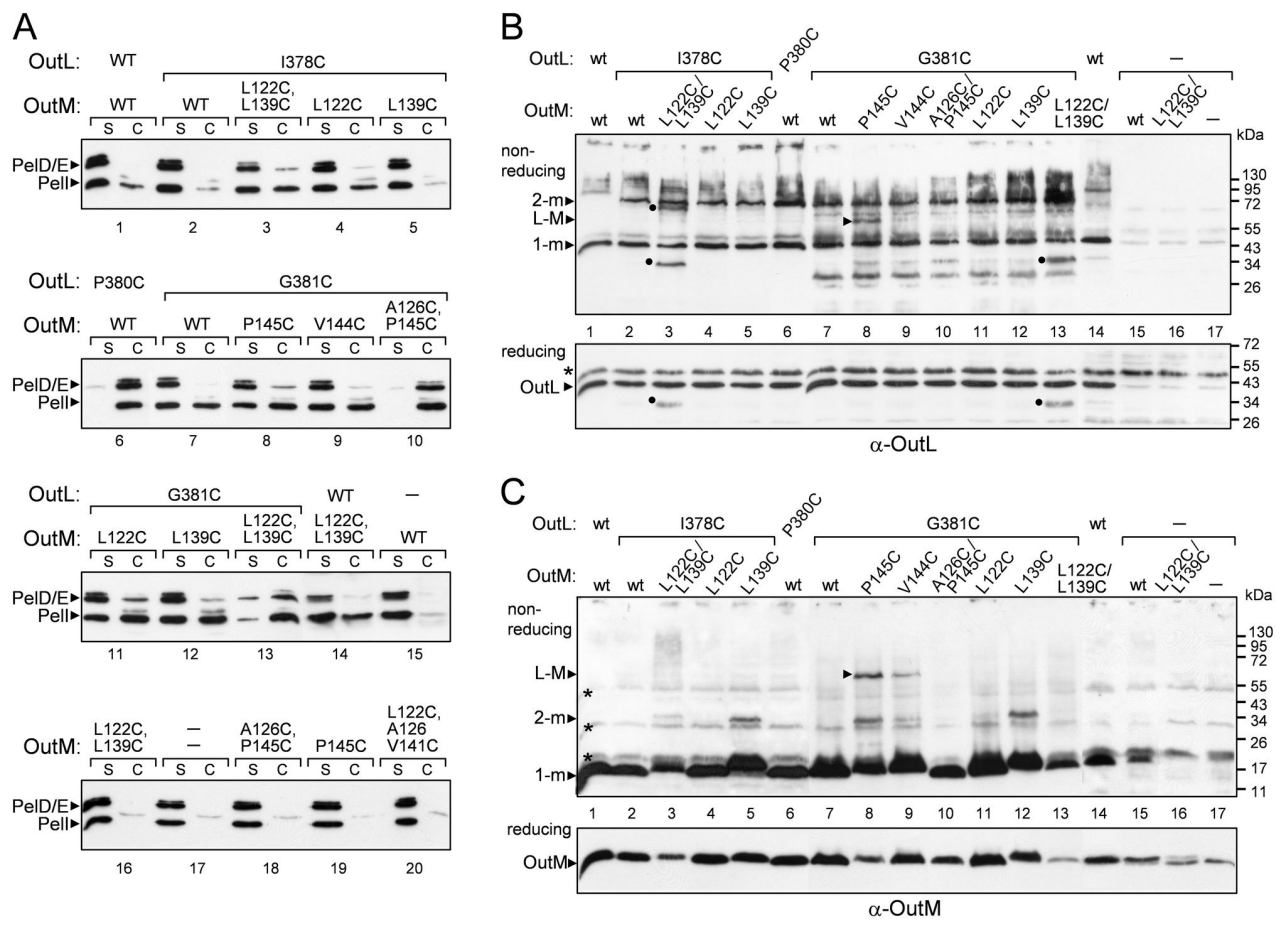
Functionality and disulfide-bonding patterns of the co-expressed cysteine variants of OutL and OutM. (*A*), secretion activity of OutL/M variants. (*B* and *C*), disulfide-bonding analysis of OutL/M variants. *D. dadantii* A4229 *wt* cells, carrying a pTdB-oLoM plasmid co-expressing mutant *outL* and *outM* alleles (indicated on top), were grown, treated and analyzed with either PelD and PelI antibodies (*A*), or with GST-OutL antibodies (*B*), or with OutM antibodies (*C*), as in Figure 4. The positions of OutL and OutM monomers (1-m), dimers (2-m) and OutL-M heterodimers (L-M) are indicated by arrowheads. Non-specific specie interacting with OutM-antibodies are shown by asterisks and OutL-degradation products, by dots. The amounts of formed dimers reflect the proximity of the respective residues from adjacent protomers.

**Figure 6:**
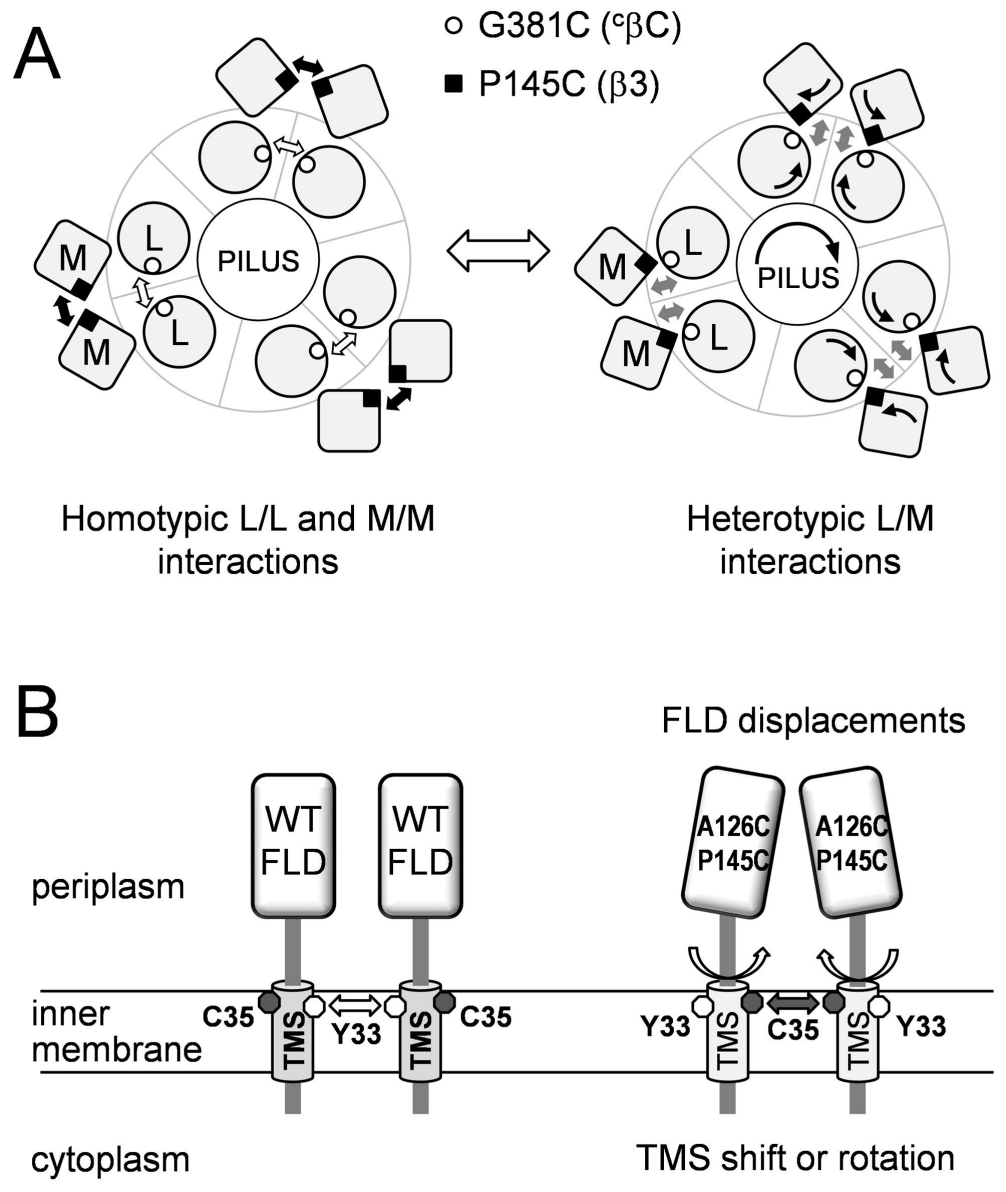
The FL domains and the TM segments of OutL and OutM are involved in the dynamic formation of functional homodimers and heterodimers. (*A*), schematic representation of the interactions between the FL domains of OutL and OutM. The view is from the periplasm inwards. The FL domains of OutL and OutM are shown as circles and squares, respectively. Left panel, adjacent FLD^OutL^ interact *via* ^c^βC strands (G381C) while adjacent FLD^OutM^ interact *via* β3 strands (P145C). Right panel, presumed interactions with an extra partner, for instance with OutG and/or OutJ/OutH pseudopilins [44,52] during pilus elongation, can switch the L/L and M/M interactions to L/M interactions. The same secondary structure elements, ^c^βC (G381C) and β3 (P145C), respectively, are involved. These displacements of the FL domains could trigger a shift or a rotation of the cognate TMS, similar to those shown in panel B. (*B*), in the wild-type OutM, the TMSs are arranged so that the Tyr33 residues of adjacent protomers are proximal (left panel). In OutM^A126C/P145C^, the displacements in the FL domains induce a rearrangement or a rotation of the TMSs such that the Cys35 residues became proximal (right panel).

 OutL^P380C^ was non-functional (Figure S2B, lane 8), suggesting that the proline substitution caused significant structural alterations. Consequently, this variant was excluded from any further analysis. To examine the presumed OutL/M interface, OutL^I378C^ and OutL^G381C^ were co-expressed in the *D. dadantii* Δ*outL* strain with one of the OutM variants, L122C, A126C, L139C, V144C, P145C, L122C/L139C or A126C/P145C. Most of the combinations restored pectinase secretion at, or near, the wild-type level except OutL^G381C^/OutM^L122C/L139C^ and OutL^G381C^/OutM^A126C/P145C^ which were more severely affected (Figure S2B, lanes 11 and 12). Surprisingly, expression of the two latter combinations in the wild-type *D. dadantii* A4229 strain also dramatically diminished pectinase secretion (Figure 5A, lanes 10 and 13). These data indicate a dominant negative interference of these variants with the secretion system. When separated in a non-reducing gel, the OutL^G381C^/OutM^P145C^ pair generated an additional species of about 60 kDa that cross-reacted with both OutL and OutM antibodies and this is consistent with an OutL-M complex (Figure 5B and C, lane 8). Such a complex was much less abundant with the OutL^G381C^/OutM^V144C^ pair (Figure 5C, lane 9) indicating that contrary to OutM^P145C^, OutM^V144C^ is not close enough to OutL^G381C^. Remarkably, when co-expressed with OutL^G381C^, OutM^P145C^ generated both hetero- and homodimers, indicating that β3-^c^βC (OutM/L) and β3-β3 (OutM/M) interactions occur simultaneously or alternate in the course of secretion (Figure 6A).

 As was observed for the homodimerization of OutM, the α2 helix also affects the presentation of P145C in the OutL/M interface. Actually, addition of the A126C mutation (α2) prevented formation of the complex by OutL^G381C^/OutM^A126C/P145C^ (Figure 5C, compare lanes 8 and 10). Similarly, a negative effect observed with OutL^G381C^/OutM^L122C/L139C^ could probably be attributed to a mutual destabilization of these variants carrying substitutions at or close to the interface. Indeed, the amount of OutM^L122C/L139C^ was significantly reduced in the presence of OutL^G381C^ and, conversely, OutL^G381C^ degradation products appeared in the presence of OutM^L122C/L139C^ (Figure 5B and C, lanes 13 and 14).

 These data support the hypothesis that the ^c^βC and β3 strands of OutL and OutM, respectively, are involved in the dynamic formation of functional homodimers and heterodimers (Figure 6A).

### Displacements of the FL domain of OutM trigger a rearrangement or rotation of the cognate TMS

 The TMS of OutM possesses three native cysteine residues namely Cys22, Cys29 and Cys35 (Figure 3E). None of these residues generated disulfide bonds during growth in culture since no dimer was detected with the wild-type OutM (Figure 4C, upper panel, lane 2). Treatment of *D. dadantii* cells with the oxidation catalyst copper phenanthroline, CoPh, which provokes disulfide bonding within the plasma membrane, generated only a low quantity of homodimers with OutM^WT^ (Figure 4C, middle panel, lane 2). This indicates that the cysteine residues in adjacent OutM TMSs are not close enough to each other. Most of the tested OutM variants were also only slightly affected by the CoPh treatment. In contrast, a significant amount of homodimer was generated with OutM^A126C/P145C^ (Figure 4C, middle panel, lane 9). Also, OutM^P145C^ no longer formed homodimers but instead generated high molecular mass species indicative of OutM oligomers (Figure 4C, lane 5). These data suggest that P145C and A126C/P145C substitutions in the FL domain alter the arrangement of the cognate TMSs such that certain membrane-embedded cysteines became proximal. To address this question, the native cysteine residues of the OutM TMS were substituted with Ile or Val. Neither the secretion nor the cross-linking patterns of the respective OutM^C22I/C29V^ and OutM^C35I^ mutants were affected; they remained mostly monomeric regardless of the CoPh treatment (Figure 4B and D, lanes 1 and 6). When C22I/C29V was combined with P145C and A126C/P145C substitutions, disulfide-bonding patterns of the resulting OutM^C22I/C29V/P145C^ and OutM^C22I/C29V/A126C/P145C^ were equivalent to those of OutM^P145C^ and OutM^A126C/P145C^, respectively (Figure 4D, upper and middle panels, compare lanes 2 to 4 and 3 to 5). This clearly indicates that neither Cys22 nor Cys29 are accessible for cross-linking. In contrast, the combination of C35I with P145C and A126C/P145C drastically altered the disulfide-bonding patterns. Unlike OutM^P145C^, the homodimer of OutM^C35I/P145C^, which was spontaneously formed *via* P145C, was still intact under CoPh treatment (Figure 4D, compare lane 7 to 4). Furthermore, OutM^C35I/A126C/P145C^ did not generate dimers even after the CoPh treatment (Figure 4D, compare lane 8 to 5). These data show that the CoPh-induced dimers of OutM^A126C/P145C^ and OutM^C22I/C29V/A126C/P145C^ were generated *via* Cys35.

 Since, in the wild-type OutM, Cys35 is not able to induce disulfide bonding, these results also indicate that P145C and A126C/P145C substitutions in the FL domain trigger a rearrangement or a rotation of the cognate TMSs, resulting in the adjacent Cys35 being close enough to form disulfide bonds (Figure 6B, right panel). To investigate this hypothesis, we probed the native arrangement of the OutM TMSs by introducing cysteine at the place of Tyr33 or Leu34 (Figure 3E). The resulting OutM^Y33C/C35I^ and OutM^L34C/C35I^ variants were fully functional (Figure 4B, lanes 9 and 10) and, contrary to OutM^WT^, they generated homodimers during CoPh treatment (Figure 4D, lanes 6, 9 and 10). The extent of dimerization was higher with Tyr33, indicating a closer TMS-TMS contact. Together, these data imply that, in adjacent OutM^WT^ protomers, the Tyr33 residues are proximal but the protomers of OutM^A126C/P145C^ are arranged such that the Cys35 residues become proximal (Figure 6B). Considering that Tyr33 and Cys35 are located on opposite sides of the TM helix (Figure 3E), this is consistent with a shift or rotation of the TMS in the mutant FLD^A126C/P145C^.

 Since the OutM^A126C/P145C^ variant remains functional (Figure 4B, lane 5), the observed displacements of its FLD and TMS could mimic some functionally relevant states of these domains. Indeed, once co-expressed with OutL, OutM^WT^ produced a significant amount of CoPh-induced homodimers (Figure S3). This suggests that OutL provokes a rearrangement of the OutM TMS similar to that observed in OutM^A126C/P145C^. Supporting this idea, the introduction of the C35I substitution in the OutL/OutM pair diminished the quantity of CoPh-induced homodimer (Figure S3). This indicates that, in the presence of OutL, the TMS of OutM are arranged such that the Cys35 residues are proximal. Therefore, it seems likely that, depending on the folding state of the FL domain of OutM or on the presence of OutL, the OutM TM helix can shift or rotate resulting in TMS-TMS contact *via* either Cys35 or Tyr33.

## Discussion

 This study investigated the organization of the inner membrane core components OutC, OutL and OutM, within the T2SS of *D. dadantii*. These components, together with the multi-spanning protein OutF and the ATPase OutE, are thought to constitute an IM platform of uncertain function and unknown stoichiometry [16]. The periplasmic regions of OutM and OutL, together with the cytoplasmic domain of OutL, have been shown to direct multiple interactions, while the TMSs of the IM components were thought to be passive anchors. However, our previous study showed that the TMS of OutC drives the self-assembly of the protein [33]. Similarly, a ToxR based two-hybrid assay has demonstrated that the TMS of EpsM (OutM homolog from *Vibrio*) can also self-dimerize *in vivo* [34]. This finding apparently contradicts previous *in vitro* results that suggested TMS is not required for EpsM dimerization [29]. Such a discrepancy may result from the different experimental approaches used. To address this question, we combined truncation analysis with *in vitro* pull-down and *in vivo* two-hybrid assays. To specifically detect the TMS-TMS interactions in the bacterial cytoplasmic membrane, we modified the bacterial two-hybrid system [40]. The TMSs of interest were fused to the periplasmic reporter protein, BlaM, indicating a correct insertion of the TMS into the IM. This technique can be used to study any TMS-TMS interaction within the natural membrane background. Overall, the results of the two-hybrid and pull-down assays are coherent and show that the TMSs of OutC, OutL and OutM are involved in multiple interactions. For example, the TMS of OutC interacts with itself and with the TMSs of OutL and OutM. Similarly, the TMS_L_ and TMS_M_ also interact with each other. These data suggest a complex interactive network within the IM.

 Mapping of the interaction sites within the periplasmic regions clearly shows that the FL domains of OutL and OutM are the main interacting modules driving the L/L, M/M and L/M interactions. These results are generally consistent with the previous yeast two-hybrid and *in vitro* studies, which identified the interaction region within the C-terminal portions of GspL and GspM [16,28,29,32,44]. These data apparently disagree with the report on the T2SS of *Vibrio* [31], which concluded that the last 66 C-terminal residues of EpsM (almost the entire FL domain) are not required for the interaction with EpsL since the remaining N-terminal portion of EpsM still interacts with EpsL. Moreover, since deletion of the 83 N-terminal residues of EpsM did not prevent its interaction with EpsL, the authors have proposed that the L/M interaction site is located between the residues 84 to 99 (α1-helix) of EpsM [31]. The present study provides an explanation for this discrepancy. We have demonstrated that two distant regions of OutL and OutM, namely TMS and FLD drive these protein interactions.

 The multi-protein two-hybrid assays indicate that homo- and heterodimerizations of the FL domains of OutL and OutM are competing that suggests an overlap of these interaction sites. This idea concurs with the structural analysis of PilO and PilN, the T4P components, orthologous to OutM and OutLp, respectively [36]. The authors have assumed that the PilO/PilO and PilN/PilO interfaces include the structural elements of the FL domains, equivalent to the α2 helix and the β3 strand of OutM. The present disulfide bonding analysis supports this hypothesis. Indeed, among the combinations of substitutions introduced in the presumed OutM^FLD^ interface, only the single L139C, V144C and P145C variants, located at or close to the β3 strand, generate a significant quantity of homodimers. These data show that the β3 strand is directly involved in the OutM^FLD^ interface. The α2 helix affects the arrangement of the β3 strands in the OutM interface since the substitutions in α2 (L122C and A126C) prevent the β3-β3 dimerization via L139C, V144C or P145C. Moreover, combination of certain substitutions in α2 and β3 (OutM^L122C/L139C^) specifically impair the protein function, suggesting important alterations in the FLD interface and/or the overall fold. However, such presumed α2-α2 or α2-β3 complexes could not be detected in this study and, consequently a direct involvement of the α2 helix in the OutM FLD interface remains uncertain.

 The generated cysteine variants of OutL efficiently produced homodimers, indicating that the OutL/L FLD interface includes the ^C^βC strand (equivalent to the β3 strand of OutM). This may indicate a similar arrangement of the FL domains of OutL and OutM within their respective homodimers. These data apparently contradict results from the structural analysis of the periplasmic region of EpsL (OutL homolog from *V. parahaemolyticus*), which assumed that the peri-EpsL dimer interface is entirely different from that of peri-EpsM and includes dissimilar structural elements, notably ^C^βA and ^C^α1 (equivalent to β1 and α1 of EpsM) [32]. In such an arrangement of peri-EpsL subunits, the ^C^βC strand (carrying the residues equivalent to those substituted in OutL) is distant from the dimer interface. However, the actual release of the PDB 2W7V shows another organization of the peri-EpsL dimer with an interface including ^C^βC and ^C^α2 (equivalent to β3 and α2 of EpsM) which is more consistent with the results of our disulfide bonding analysis.

 The present study demonstrates the dynamic nature of the FLD-FLD interactions between OutL and OutM. Indeed, OutM^P145C^ generated not only homodimers but also heterodimers with OutL^G381C^. In turn, OutL^G381C^ was also efficiently homodimerized. These data imply a succession of homotypic β3-β3 (P145C-P145C) and ^c^βC-^c^βC (G381C-G381C) interactions of adjacent OutM/M and OutL/L protomers, respectively, and heterotypic β3-^c^βC (P145C-G381C) interactions between OutM and OutL (Figure 6A). These results are consistent with our previous study which showed alternating interactions between the periplasmic domains of OutC and OutD [15]. More unexpectedly, the present work suggests that the successive FLD-FLD contacts mediate rearrangements and/or rotations of the cognate TMSs. Indeed, depending on the presence of a particular mutation in the FLD^OutM^ (A126C/P145C) or an appropriate interacting partner (OutL), the TMSs of adjacent OutM subunits can move or rotate resulting in TMS-TMS contact *via* either Cys35 or Tyr33 (Figure 6B). This finding provides a simple mechanistic explanation of how the same face of the FLD^OutM^, including β3, can move from homotypic (M-M) to heterotypic (M-L) contacts. In fact, the FLD can act as a pivot and its successive movements induce certain coordinated rotations of the cognate TMS. Considering the number of other assumed TMS-TMS interactions, the inner membrane platform of the T2SS could constitute a sort of cogwheel mechanism, transmitting the signal from the periplasmic to the cytoplasmic side of the secretion machinery including the ATPase OutE or *vice versa*. Consistent with this idea, a previous study has shown that activation of the ATPase EpsE necessitates an interaction between the EpsL segment adjacent to the TMS and the membrane lipids [45]. This would lead to a coordinated movement of the cognate TMS_L_ and, hence, it is consistent with a dynamic arrangement of the TMSs within the IM platform of T2SS. Remarkably, a similar cysteine-scanning/bonding approach has been previously used with several other cell machineries and revealed dynamic nature of these transmembrane complexes [42,46-48]. 

 However, an important question remains to be elucidated, that of determining what events might cause successive FLD-FLD interactions. One possibility could be an interaction with another T2SS component or with the secretion substrate. The periplasmic domain of OutL has been shown to interact with those of the pseudopilins OutJ and OutH [44], which are thought to be part of the pilus tip complex [49-51]. A recent study has also demonstrated direct contact between EpsL and the major pseudopilin EpsG, homologues to OutL and OutG, respectively [52]. Thus, we can imagine that each step of pilus elongation would, in turn, switch the interactions of the OutL FLD, L/L to L/M or inversely (Figure 6A). Our previous studies have shown that the periplasmic HR domain of OutC interacts with several sites within the periplasmic domains of the secretin OutD [14,15]. These interactions could control the entry of the secretion substrate into the periplasmic vestibule formed by the secretin and its gating/opening. In addition, the OutL/OutM couple significantly increases the stability of OutC and alters its interactions with OutD [15], indicating direct interaction of OutL/OutM with OutC. The present study suggests that the periplasmic region of OutC does not interact with those of OutL and OutM, but that their TMSs interact. Therefore, the interactions sensed by the periplasmic domains of OutC could be transmitted, *via* the TMS, to OutL and OutM, or *vice versa*. 

Clearly, further studies are needed to elucidate the exact molecular mechanisms of the TMS assembly and the details of the interactions within the IM portion of the T2SS machinery.

## Materials and Methods

### Strains, plasmids and growth conditions

 The bacterial strains and plasmids used in this study are listed in Tables S1 and S2. The bacteria were grown in Luria-Bertani (LB) at 28°C with shaking at 120 rpm. If necessary, antibiotics were added at the following final concentrations: ampicillin, 150 µg.ml^-1^; kanamycin, 100 µg.ml^-1^ and chloramphenicol, 50 µg.ml^-1^.

 DNA cloning and manipulation were carried out using standard methods. Site-directed mutagenesis was performed with the QuickChange kit (Stratagene) and the primers listed in Table S3. The sequences of mutant and amplified genes were checked (Eurofins MWG Operon). Plasmids pTdB-oM and pTdB-oLoM, expressing *outM* or *outL-outM* genes were constructed by cloning the corresponding DNA fragments under the control of P*pelC*. To generate plasmids expressing Out derivatives fused to either GST, 6His, T18, or T25, the corresponding *out* gene fragments were amplified by PCR and subcloned into either pGEX-6P-3, pET20b(+), pUT18C or pKT25 vectors, respectively. More details are described in File S1. The *D. dadantii* Δ*outL* and *outM* mutant strains were constructed as described in File S1.

### Functional tests and *in vivo* disulfide cross-linking analysis

 To assess the functional relevance of the generated cysteine substitutions in OutM and OutL, the mutant alleles were introduced into pTdB-oM and/or pTdB-oLoM plasmid and expressed in *D. dadantii* A5269 *outM* or A5434 *∆outL pecS* strain, respectively. The bacteria were grown in LB supplemented with ampicillin at 150 µg.ml^-1^ and, if necessary, with galacturonate, 1 g.l^-1^, aerobically at 120 rpm and at 28°C for 14 h to steady-state. Culture supernatant and cells were separated at 10,000 g for 2 min and analyzed by immunoblotting with PelD and PelI-antibodies [33]. 

 To assess the extent of disulfide cross-linking between the periplasmic domains, the spontaneous formation of disulfide bonds in steady-state cultures was examined. To generate formation of disulfide bonds between the TMSs within the plasma membrane, cells were incubated with the oxidation catalyst copper phenanthroline, CoPh [53]. Briefly, bacteria were grown as above and cells from 1.3 ml of culture (OD_600_ of 1.6-1.8) were spun at 10,000 g for 1 min and washed with TBS (50 mM Tris-HCl pH 7.5, 100 mM NaCl). Next, the cells were resuspended in 1 ml of TBS and divided into two aliquots. One aliquot was supplemented with CoPh to 1 mM and incubated for 10 min at 15°C. After that, the cells were washed in TBS and, in order to block the free thiol groups and prevent further disulfide bond formation, they were incubated with 10 mM iodoacetamide in TBS for an additional 30 min at 15°C. Another aliquot was immediately supplemented with 10 mM iodoacetamide in TBS and incubated for 30 min at 15°C. Then, the cells were spun, washed, resuspended in 100 μl Laemmli sample buffer without 2-mercaptoethanol and lysed in boiling water for 10 min. The samples were next supplemented with the same volume of 12 M urea (at 55°C), incubated for 20 min at 37°C and loaded onto 9 % Tris-Tricine SDS-PAGE [54] supplemented with 6 M urea. The extent of disulfide bonding was assessed by immunoblotting with OutM or GST-OutL antibodies. To estimate the abundance of the OutM and OutL variants in the cells, the same cell samples were treated with 10 mM 2-mercaptoethanol.

### Bacterial two-hybrid assays

 The bacterial two-hybrid system [40], kindly provided by G. Karimova, was used as described previously [33]. Combinations of various Out derivatives fused to T18 or T25 (listed in Table S1) were co-transformed into the *Escherichia coli cyaA* strain DHP1 [40] and the transformants were plated on MacConkey-maltose agar. The color of the colonies was monitored during an incubation at 30°C for 36-48 h. To quantify the protein interactions, DHP1 cells, carrying various plasmid combinations, were grown in LB supplemented with 1 mM isopropyl-β-*D*-thiogalactopyranoside and with appropriate antibiotics at 28°C for 18 h and then used for β-galactosidase assays, as described [33]. Cells were permeabilized by adding 50 μl toluene to 1 ml of culture and vortexing 3 times for 15 sec with 10 min intervals. Then, 20-100 μl of cell extracts were added to 880-800 μl of Z buffer [55] and incubated at 37°C for 5 min. The reaction was started by adding 100 μl of ortho-Nitrophenyl-β-galactoside (4 mg/ml) and stopped by addition of 0.5 ml of 1 M Na_2_CO_3_. The reaction time, the OD_420_ of the reaction mixture and OD_600_ of the initial cultures were recorded and used to calculate the enzymatic activity. Next, the values of β-galactosidase (in Miller units [55]) observed with each tested plasmid pair were expressed as percentage of that with the full-length T18-OutC/T25-OutC, considered as 100% (positive control). All assays were performed from triplicate cultures on three to four different bacterial transformants and on several different days. Protease accessibility assay was performed as described [33].

### Pull-down assays

 The Out proteins fused to GST or 6His were produced in *E. coli* BL21(DE3) carrying an appropriate pGEX-6P-3 or pET20b(+) plasmid as described [13]. His-tagged derivatives were purified by nickel-affinity chromatography in the presence of 1% (v/v) Triton X-100. Alternatively, His-OutL and OutM were exclusively labeled with [^35^S] cysteine-methionine (PerkinElmer) and directly extracted from the whole cells in the presence of 1% (v/v) Triton X-100, as described [56]. GST pull-down assays were performed in the presence of 1% Triton X-100 at 15°C [13]. Briefly, equal amounts of various GST-Out derivatives were immobilized on Glutathione Sepharose. Then, an appropriate prey protein (His-Out derivative) was incubated for 1 h with immobilized GST-baits and unbound proteins were washed out for 5 times. The bound proteins were eluted with Laemmli sample buffer, separated by SDS-PAGE and probed by immunoblotting with antibodies raised against OutC, OutM or GST-OutL, or with Ni-NTA-conjugated with peroxidase (Qiagen). [^35^S]-labeled proteins were revealed by autoradiography.

### Molecular modeling

 C-terminal regions of OutL (residues 311 to 400) and OutM (residues 83 to 162) were modeled with the homology molecular modeling program MODELLER 9v11 [57]. For OutL, the software identified the following crystal structure as template allowing a very confident modeling of the studied region: PDB entry 2W7V (periplasmic domain of EpsL from the type 2 secretion system of *V. parahaemolyticus*). For OutM, a unique structure was used as template as well (PDB entry 1UV7: periplasmic domain of EpsM from *V. cholerae*). For both modeling studies, 30 distinct models have been generated, and their geometry was assessed by a Ramachandran plot calculated with the program PROCHECK [58]. Consequently, the most satisfying model was retained. For OutL, it has 96.3% of non-proline and non-glycine residues in the most favored regions, 3.7% in additionally allowed regions and none in disallowed regions. For OutM, Ramachandran plot values were 97.2% residues in most favored regions, 2.8% in additionally allowed regions and none in disallowed regions.

## Supporting Information

File S1 (PDF)

Figure S1 **Schematic representation of OutC (*A*), OutL (*B*) and OutM (*C*) and their derivatives used in this study.** The positions of various domains are indicated with grey boxes: TMS, transmembrane segment (TMHMM Server v2.0 [59]); HR, homology region domain (PDB entry 2LNV, [14]); PDZ domain (PDB entry 2I6V, [11]); N1, N2 and N3, cytoplasmic OutL domains (PDB entry 1W97, [30]); FL, ferredoxin-like domains of OutL (PDB entry 2W7V, [32]) and OutM (PDB entry 1UV7, [29]).
(PDF)

Figure S2 **Disulfide-bonding patterns and functionality of cysteine variants of OutL and OutM co-expressed in *D. dadantii* A5434Δ*outL*.** (*A*), disulfide-bonding analysis of OutL/M variants. (*B* and *C*), secretion activity of OutL/M variants. *D. dadantii* A5434Δ*outL* *pecS* cells, carrying a pTdB-oLoM plasmid co-expressing mutant *outL* and *outM* alleles (indicated on top), were grown, treated and analyzed with either GST-OutL antibodies (*A*), or PelD and PelI antibodies (*B*), as in Figure 4. The positions of OutL monomers (1-m) and dimers (2-m) are indicated by arrowheads. Non-specific specie interacting with GST-OutL antibodies are shown by asterisks. In (*A*), the amounts of formed dimers reflect the proximity of the respective residues from adjacent protomers. In (*B*), the quantity of secreted proteins present in the culture supernatant reflects the efficiency of secretion.
(PDF)

Figure S3 **Co-expression of OutL with OutM provokes a rearrangement of the OutM TMS.** Disulfide-bonding analysis of OutL/M variants. *D. dadantii* A4229 cells, carrying either a pTdB-oM or a pTdB-oLoM plasmid expressing indicated *outL* and *outM* variants (on top), were grown to steady state and were either directly treated with iodoacetamide (CoPh -), to block any remaining free thiol groups or were firstly incubated with the oxidation catalyst (CoPh +) before the iodoacetamide treatment. The extent of disulfide bonding was assessed using non-reducing SDS-PAGE, followed by immunoblotting with OutM-antibodies. The positions of OutM monomers (1-m) and dimers (2-m) are indicated by arrowheads. Non-specific specie interacting with OutM antibodies are shown by asterisks. The amounts of dimers reflect the proximity of the respective residues from adjacent protomers.
Note that the co-expression of OutL with OutM provoked a significant increase in the quantity of OutM (compare lanes 1 and 2 to 3 and 4) and also a CoPh-induced homodimerization of OutM (compare lanes 6 to 7). The two right lanes (shaded) were overexposed to better show the absence of dimer of OutM^WT^. The introduction of the C35I substitution in the OutL/OutM pair diminished the quantity of CoPh-induced homodimer (compare lanes 7 to 8).
(PDF)

Table S1 **Bacterial strains and plasmids used in this study.**
(PDF)

Table S2 **Plasmids expressing cysteine variants of OutL and OutM used in this study.**
(PDF)

Table S3 **Primers employed in the study.**
(PDF)
