# Supplementary material for: Dynamic Interplay between the Periplasmic and Transmembrane Domains of GspL and GspM in the Type II Secretion System
Source: PLoS One. 2013 Nov 1;8(11):e79562. doi: 10.1371/journal.pone.0079562 (PMC3815138; doi:10.1371/journal.pone.0079562)
Supplement: File S1 — (PDF) [file pone.0079562.s001.pdf]

Plasmid construction

To create pET-oM plasmid producing 6HisOutM, the *outM* gene fragment was amplified using the OuMHis and OuMER primers and cloned into the *Nde*I/*Eco*RI sites of pET20b(+). To generate pTdB-oM expressing *6His-outM*, under the control of P*pelC*, the *Bgl*II/*Eco*RI *outM* gene fragment preceded by an RBS was subcloned from pET-oM into the *Bam*HI/*Eco*RI sites of pTdB-oC in the place of *outC*. pET-oL and pTdB-oL plasmids were constructed by a similar way using the OuLHis and OuLER primers. To create pTdB-oLoM plasmid, co-expressing *outL-outM*, the *outL-M* gene fragment was amplified using the OuLHis and OuMER primers and cloned as above.

To generate T18/T25-Out fusions, an appropriate *out* gene fragment was amplified by PCR and fused in frame to the 3'-end of *cya* gene fragments coding for either T18 or T25 domain of adenylate cyclase on the plasmids pUT18C and pKT25, respectively (Table S1). To generate T25/T18-TMS-BlaM fusions, the *Pvu*II/*Eco*RI *'blaM* gene fragment (Broome-Smith *et al.*, 1990) was in addition fused to the 3'-end of the out_{TMS} gene fragment (Table S1 and Fig. 2A, top right). To create pKT-GST-oLp plasmid expressing *T25-gst-'outLp* fusion, the *gst-outLp* gene fragment was subcloned from pGX-oLp plasmid into pKT-GST-oC_{40-272} plasmid [13] by using the *Sfu*I/*Eco*RI sites. To co-express the T25-OutLper fusion and the isolated OutMper region (pKT-oLp+Mp), the *'outM* fragment coding for $OutM_{37-162}$ was cloned in a natural-like way, such that the beginning of *outMp* overlapped the translation stop of the *cya-outLp* fusion and the natural *outMp* RBS is located within the *outLp* coding region. Precisely, the *outLp* fragment was amplified using the OuLNhe and RCOuLNdRI primers while the *outMp* fragment was amplified using the OuMNde5' and OuMER primers and the two fragments were linked together *via* the generated *Nde*I sites (3' of *outLp* and 5' of *outMp*) and then cloned into the *Xba*I/*Eco*RI sites of pKT25.

Strain construction

The *D. dadantii* Δ*outL* A3696 strain, carrying a deletion within the chromosomic *outL* allele, was constructed by marker exchange-eviction mutagenesis, as described previously [4]. Briefly, the *D. dadantii* A3688 strain, sucrose-sensitive and secretion-deficient since it carries the *nptI-sacB-sacR* (Km^R) cartridge into the chromosomic *outL*, was transformed with a pTdB-doL plasmid bearing an in frame deletion within the *outL* gene. Then, the mutant allele was exchanged for the chromosomal allele by selecting for sucrose tolerance and sensitivity to kanamycin. To construct *D. dadantii outM::cat* A5269 strain, first, a *Bam*HI site was introduced into the *outM* gene using OuMBH2 and ROuMBH2 primers (Table S3). Next, the CmR gene cartridge was inserted into this site of *outM* onto the pTdB-oLoM plasmid and then, the *outM::cat* allele was introduced into the chromosome by gene exchange recombination. A correct recombination of the mutant alleles into the chromosome was checked by PCR.

Broome-Smith JK, Tadayyon M, Zhang Y (1990) Beta-lactamase as a probe of membrane protein assembly and protein export. Mol Microbiol 4: 1637–1644.
