# Supplementary material for: Dynamic Interplay between the Periplasmic and Transmembrane Domains of GspL and GspM in the Type II Secretion System
Source: PLoS One. 2013 Nov 1;8(11):e79562. doi: 10.1371/journal.pone.0079562 (PMC3815138; doi:10.1371/journal.pone.0079562)
Supplement: Figure S1 — Schematic representation of OutC (A), OutL (B) and OutM (C) and their derivatives used in this study. The positions of various domains are indicated with grey boxes: TMS, transmembrane segment (TMHMM Server v2.0 [59]); HR, homology region domain (PDB entry 2LNV, [14]); PDZ domain (PDB entry 2I6V, [11]); N1, N2 and N3, cytoplasmic OutL domains (PDB entry 1W97, [30]); FL, ferredoxin-like domains of OutL (PDB entry 2W7V, [32]) and OutM (PDB entry 1UV7, [29]). (PDF) [file pone.0079562.s002.pdf]

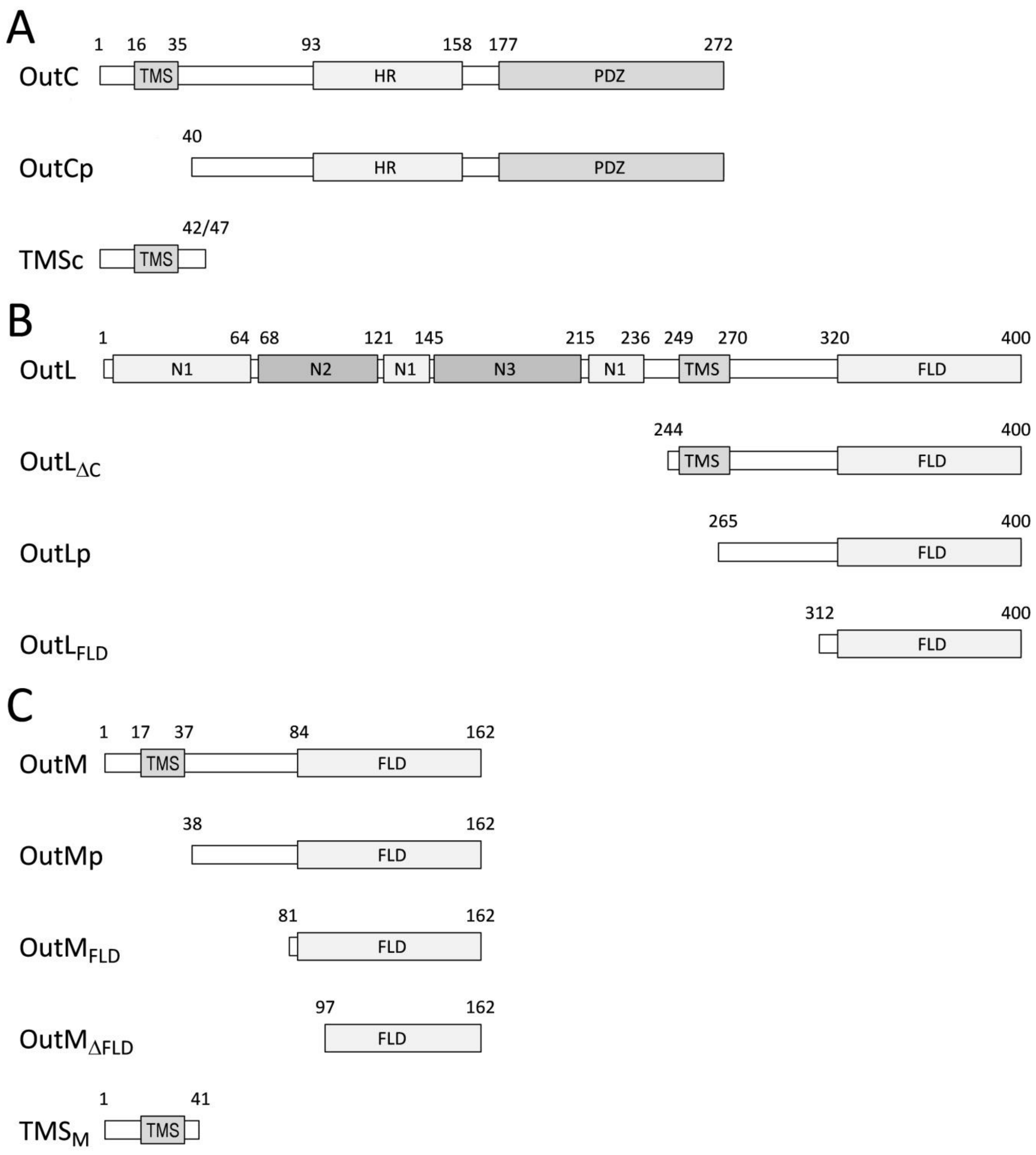

Figure S1. Schematic representation of OutC (*A*), OutL (*B*) and OutM (*C*) and their derivatives used in this study. The positions of various domains are indicated with grey boxes: TMS, transmembrane segment (TMHMM Server v2.0 [59]); HR, homology region domain (PDB entry 2LNV, [14]); PDZ domain (PDB entry 2I6V, [11]); N1, N2 and N3, cytoplasmic OutL domains (PDB entry 1W97, [30]); FL, ferredoxin-like domains of OutL (PDB entry 2W7V, [32]) and OutM (PDB entry 1UV7, [29]).
