# Supplementary material for: Dynamic Interplay between the Periplasmic and Transmembrane Domains of GspL and GspM in the Type II Secretion System
Source: PLoS One. 2013 Nov 1;8(11):e79562. doi: 10.1371/journal.pone.0079562 (PMC3815138; doi:10.1371/journal.pone.0079562)
Supplement: Figure S2 — Disulfide-bonding patterns and functionality of cysteine variants of OutL and OutM co-expressed in D. dadantii A5434ΔoutL. (A), disulfide-bonding analysis of OutL/M variants. (B and C), secretion activity of OutL/M variants. D. dadantii A5434ΔoutL pecS cells, carrying a pTdB-oLoM plasmid co-expressing mutant outL and outM alleles (indicated on top), were grown, treated and analyzed with either GST-OutL antibodies (A), or PelD and PelI antibodies (B), as in Figure 4. The positions of OutL monomers (1-m) and dimers (2-m) are indicated by arrowheads. Non-specific specie interacting with GST-OutL antibodies are shown by asterisks. In (A), the amounts of formed dimers reflect the proximity of the respective residues from adjacent protomers. In (B), the quantity of secreted proteins present in the culture supernatant reflects the efficiency of secretion. (PDF) [file pone.0079562.s003.pdf]

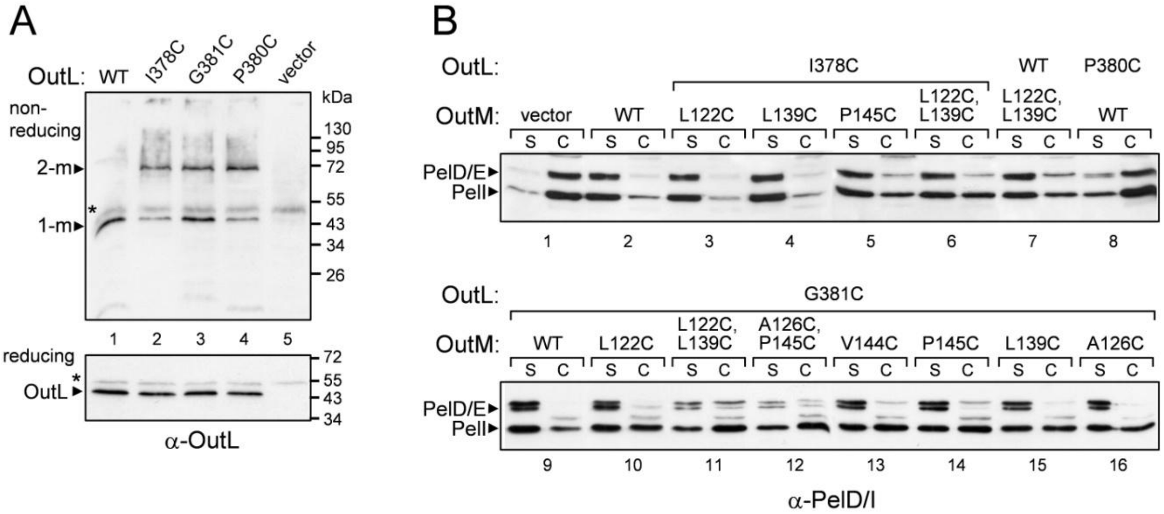

Figure S2. Disulfide-bonding patterns and functionality of cysteine variants of OutL and OutM co-expressed in *D. dadantii* A5434Δ*outL*. (*A*), disulfide-bonding analysis of OutL/M variants. (*B* and *C*), secretion activity of OutL/M variants. *D. dadantii* A5434Δ*outL pecS* cells, carrying a pTdB-oLoM plasmid co-expressing mutant *outL* and *outM* alleles (indicated on top), were grown, treated and analyzed with either GST-OutL antibodies (*A*), or PelD and PelI antibodies (*B*), as in Fig. 4. The positions of OutL monomers (1-m) and dimers (2-m) are indicated by arrowheads. Non-specific specie interacting with GST-OutL antibodies are shown by asterisks. In (*A*), the amounts of formed dimers reflect the proximity of the respective residues from adjacent protomers. In (*B*), the quantity of secreted proteins present in the culture supernatant reflects the efficiency of secretion.
