# Supplementary material for: Dynamic Interplay between the Periplasmic and Transmembrane Domains of GspL and GspM in the Type II Secretion System
Source: PLoS One. 2013 Nov 1;8(11):e79562. doi: 10.1371/journal.pone.0079562 (PMC3815138; doi:10.1371/journal.pone.0079562)
Supplement: Figure S3 — Co-expression of OutL with OutM provokes a rearrangement of the OutM TMS. Disulfide-bonding analysis of OutL/M variants. D. dadantii A4229 cells, carrying either a pTdB-oM or a pTdB-oLoM plasmid expressing indicated outL and outM variants (on top), were grown to steady state and were either directly treated with iodoacetamide (CoPh -), to block any remaining free thiol groups or were firstly incubated with the oxidation catalyst (CoPh +) before the iodoacetamide treatment. The extent of disulfide bonding was assessed using non-reducing SDS-PAGE, followed by immunoblotting with OutM-antibodies. The positions of OutM monomers (1-m) and dimers (2-m) are indicated by arrowheads. Non-specific specie interacting with OutM antibodies are shown by asterisks. The amounts of dimers reflect the proximity of the respective residues from adjacent protomers. Note that the co-expression of OutL with OutM provoked a significant increase in the quantity of OutM (compare lanes 1 and 2 to 3 and 4) and also a CoPh-induced homodimerization of OutM (compare lanes 6 to 7). The two right lanes (shaded) were overexposed to better show the absence of dimer of OutMWT. The introduction of the C35I substitution in the OutL/OutM pair diminished the quantity of CoPh-induced homodimer (compare lanes 7 to 8). (PDF) [file pone.0079562.s004.pdf]

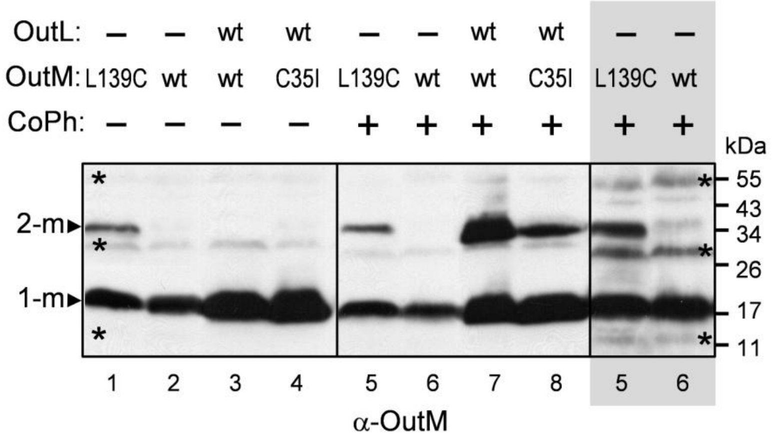

Figure S3. Co-expression of OutL with OutM provokes a rearrangement of the OutM TMS.

Disulfide-bonding analysis of OutL/M variants. *D. dadantii* A4229 cells, carrying either a pTdB-oM or a pTdB-oLoM plasmid expressing indicated *outL* and *outM* variants (on top), were grown to steady state and were either directly treated with iodoacetamide (CoPh -), to block any remaining free thiol groups or were firstly incubated with the oxidation catalyst (CoPh +) before the iodoacetamide treatment. The extent of disulfide bonding was assessed using non-reducing SDS-PAGE, followed by immunoblotting with OutM-antibodies. The positions of OutM monomers (1-m) and dimers (2-m) are indicated by arrowheads. Non-specific specie interacting with OutM antibodies are shown by asterisks. The amounts of dimers reflect the proximity of the respective residues from adjacent protomers.

Note that the co-expression of OutL with OutM provoked a significant increase in the quantity of OutM (compare lanes 1 and 2 to 3 and 4) and also a CoPh-induced homodimerization of OutM (compare lanes 6 to 7). The two right lanes (shaded) were overexposed to better show the absence of dimer of $OutM^{WT}$. The introduction of the C35I substitution in the OutL/OutM pair diminished the quantity of CoPh-induced homodimer (compare lanes 7 to 8).
