# Supplementary material for: Dynamic Interplay between the Periplasmic and Transmembrane Domains of GspL and GspM in the Type II Secretion System
Source: PLoS One. 2013 Nov 1;8(11):e79562. doi: 10.1371/journal.pone.0079562 (PMC3815138; doi:10.1371/journal.pone.0079562)
Supplement: Table S1 — Bacterial strains and plasmids used in this study. (PDF) [file pone.0079562.s005.pdf]

Table S1. Bacterial strains and plasmids used in this study

Strain	Genotype/phenotype	Reference
Escherichia coli		
BL21(DE3)	F$^-$ *dcm ompT hsdS*B(r$_B^-$, m$_B^-$) *gal lon* λ(DE3)	Stratagene
DHP1	F$^-$ *cya glnV44*(*AS*) *recA1 endA1 gyrA96* (*Nal*^{R}) *thi1* *hsdR17 spoT1 rfbD1*	[40]
NM522	*supE thi-1* Δ(*lac-proAB*) Δ(*mcrB-hsdSM*)*5* (r_K^- m_K^+) [F′ *proAB lacI*^{q}ZΔ*M15*]	Stratagene
Dickeya dadantii		
A3688	*rafR ganB outL::nptI-sacB-sacR* (Km^R)	G. Condemine
A3696	*rafR ganB* Δ*outL*	G. Condemine
A4229	*rafR ganB*	Laboratory collection
A5434	*rafR ganB* Δ*outL pecS::cat* (Cm^R)	This work
A5269	*rafR ganB outM::cat* (Cm^R)	This work
Plasmids		
pTdB-oM [a]	pT7-6 carrying *6His-outM* under P*pelC*	This work
pTdB-oL	pT7-6 carrying *6His-outL* under P*pelC*	This work
pTdB-oLoM [a]	pT7-6 co-expressing *outL* and *outM* under P*pelC*	This work
Vectors expressing GST and 6His derivatives		
pET-20b(+)	Signal sequence and 6His-tag fusion vector, Ap^R	Novagen
pQE32	6His-tag fusion vector, Ap^R	Qiagen
pGEX-6P-3	GST-fusion vector with PreScission protease site, Ap^R	GE Healthcare
pQE-oC	pQE32 carrying *6his-outC* (aa 2 to 272)	[4]
pGX-oC	pGEX-6P-3 carrying *gst-outC* (aa 2 to 272)	[33]
pGX-oCp	pGEX-6P-3 carrying *gst-outC'* (aa 40 to 272)	[33]
pGX-oC$_{TMS}$	pGEX-6P-3 carrying *gst-outC'* (aa 2 to 42)	[33]
pET-oM	pET-20b(+) carrying *6his-outM* (aa 2 to 162)	This work
pGX-oM	pGEX-6P-3 carrying *gst-outM* (aa 2 to 162)	This work
pGX-oMp	pGEX-6P-3 carrying *gst-outM'* (aa 38 to 162)	This work
pGX-oM$_{TMS}$	pGEX-6P-3 carrying *gst-outM'* (aa 2 to 37)	[13]
pET-oL	pET-20b(+) carrying *6his-outL* (aa 2 to 400)	This work
pGX-oL	pGEX-6P-3 carrying *gst-outL* (aa 2 to 400)	This work
pGX-oLp	pGEX-6P-3 carrying *gst-'outL* (aa 264 to 400)	This work
pGX-oLΔp	pGEX-6P-3 carrying *gst-outL'* (aa 1 to 265)	This work
Two-hybrid vectors		
pKT25	pSU40 derivative coding T25 fragment of CyaA, Kn^R	[40]
pUT18C	pUC19 derivative coding T18 fragment of CyaA, Ap^R	[40]
pUT18CCm	pUT18C carrying *cat* in place of *blaM*, Cm^R	This work
pKT-oC	pKT25 carrying *T25-outC* (aa 2 to 272)	[33]
pKT-oCp	pKT25 carrying *T25-'outC* (aa 40 to 272)	[33]
pKT-C$_{TMS}$-Bla	pKT25 carrying *T25-outC'-blaM* (aa 2 to 47)	This work
pUT-oC	pUT18C carrying *T18-outC* (aa 2 to 272)	[33]

pUT-oCp	pUT18C carrying *T18*-'*outC* (aa 40 to 272)	[33]
pUT-C_{TMS}-Bla	pUT18C carrying *T18-outC'-blaM* (aa 2 to 47)	This work
pKT-oLΔc	pKT25 carrying *T25-'outL* (aa 244 to 400)	This work
pKT-oLp	pKT25 carrying *T25*-'*outL* (aa 265 to 400)	This work
pKT-oL_{FLD}	pKT25 carrying *T25*-'*outL* (aa 312 to 400)	This work
pKT-oLp+Mp	pKT25 coexpressing *T25*-'*outL* (aa 265 to 400) and '*outM* (aa 38 to 162)	This work
pKT-GST-oLp	pKT25 carrying *T25-gst-'outL* (aa 265 to 400)	This work
pKT-L_{TMS}-Bla	pKT25 carrying *T25-outL'-blaM* (aa 244 to 273)	This work
pUT-oLΔc	pUT18C carrying *T18-'outL* (aa 244 to 400)	This work
pUT-oLp	pUT18C carrying *T18*-'*outL* (aa 265 to 400)	This work
pUT-oL_{FLD}	pUT18C carrying *T18*-'*outL* (aa 312 to 400)	This work
pUT-oLp+Mp	pUT18C coexpressing *T18*-'*outL* (aa 265 to 400) and '*outM* (aa 38 to 162)	This work
pUT-L_{TMS}-Bla	pUT18C carrying *T18-outL'-blaM* (aa 244 to 273)	This work
pKT-oM	pKT25 carrying *T25-'outM* (aa 3 to 162)	This work
pKT-oMp	pKT25 carrying *T25*-'*outM* (aa 38 to 162)	This work
pKT-oM_{FLD}	pKT25 carrying *T25*-'*outM* (aa 81 to 162)	This work
pKT-M_{TMS}-Bla	pKT25 carrying *T25-outM'-blaM* (aa 3 to 41)	This work
pUT-oM	pUT18C carrying *T18-'outM* (aa 3 to 162)	This work
pUT-oMp	pUT18C carrying *T18*-'*outM* (aa 38 to 162)	This work
pUT-oM_{FLD}	pUT18C carrying *T18*-'*outM* (aa 81 to 162)	This work
pUT-$oM_{\Delta FLD}$	pUT18C carrying *T18*-'*outM* (aa 97 to 162)	This work
pUT-M_{TMS}-Bla	pUT18C carrying *T18-outM'-blaM* (aa 3 to 41)	This work
pUT-Tet_{TMS}-Bla	pUT18C carrying *T18-tetA'-blaM* (aa 4 to 34)	This work

[a] Plasmids expressing cysteine variants of OutC and OutD are listed in the supplemental Table S2.
