# Supplementary material for: Dynamic Interplay between the Periplasmic and Transmembrane Domains of GspL and GspM in the Type II Secretion System
Source: PLoS One. 2013 Nov 1;8(11):e79562. doi: 10.1371/journal.pone.0079562 (PMC3815138; doi:10.1371/journal.pone.0079562)
Supplement: Table S2 — Plasmids expressing cysteine variants of OutL and OutM used in this study. (PDF) [file pone.0079562.s006.pdf]

Table S2. Plasmids expressing cysteine variants of OutL and OutM used in this study

Plasmid	Expressed genes	Reference
Vectors expressing 6His-OutM variants under the control of P*pelC*		
pTdB-oM$_{L122C}$	*outM* (L122C)	This work
pTdB-oM$_{A126C}$	*outM* (A126C)	This work
pTdB-oM$_{L139C}$	*outM* (L139C)	This work
pTdB-oM$_{V141C}$	*outM* (V141C)	This work
pTdB-oM$_{V144C}$	*outM* (V144C)	This work
pTdB-oM$_{P145C}$	*outM* (P145C)	This work
pTdB-oM$_{C22I/C29V}$	*outM* (C22I/C29V)	This work
pTdB-oM$_{C35I}$	*outM* (C35I)	This work
pTdB-oM$_{C22I/C29V/P145C}$	*outM* (C22I/C29V/P145C)	This work
pTdB-oM$_{C22I/C29V/A126C/P145C}$	*outM* (C22I/C29V/A126C/P145C)	This work
pTdB-oM$_{C35I/P145C}$	*outM* (C35I/P145C)	This work
pTdB-oM$_{C35I/A126C/P145C}$	*outM* (C35I/A126C/P145C)	This work
pTdB-oM$_{L122C/A126C}$	*outM* (L122C/A126C)	This work
pTdB-oM$_{L122C/L139C}$	*outM* (L122C/L139C)	This work
pTdB-oM$_{L122C/V141C}$	*outM* (L122C/V141C)	This work
pTdB-oM$_{L122C/V144C}$	*outM* (L122C/V144C)	This work
pTdB-oM$_{L122C/P145C}$	*outM* (L122C/P145C)	This work
pTdB-oM$_{A126C/L139C}$	*outM* (A126C/L139C)	This work
pTdB-oM$_{A126C/V144C}$	*outM* (A126C/V144C)	This work
pTdB-oM$_{A126C/P145C}$	*outM* (A126C/P145C)	This work
pTdB-oM$_{L122C/A126C/V141C}$	*outM* (L122C/A126C/V141C)	This work
pTdB-oM$_{L122C/A126C/P145C}$	*outM* (L122C/A126C/P145C)	This work
Vectors coexpressing OutL and OutM variants under the control of P*pelC*		
pTdB-oLoM$_{L122C/L139C}$	*outL*(WT) and *outM*(L122C/L139)	This work
pTdB-oL$_{I378C}$oM	*outL*(I378C) and *outM*(WT)	This work
pTdB-oL$_{P380C}$oM	*outL*(P380C) and *outM*(WT)	This work
pTdB-oL$_{G381C}$oM	*outL*(G381C) and *outM*(WT)	This work
pTdB-oL$_{I378C}$oM$_{L122C}$	*outL*(I378C) and *outM*(L122C)	This work
pTdB-oL$_{I378C}$oM$_{A126C}$	*outL*(I378C) and *outM*(A126C)	This work
pTdB-oL$_{I378C}$oM$_{L139C}$	*outL*(I378C) and *outM*(L139C)	This work
pTdB-oL$_{I378C}$oM$_{L122C/L139C}$	*outL*(I378C) and *outM*(L122C/L139)	This work
pTdB-oL$_{I378C}$oM$_{V144C}$	*outL*(I378C) and *outM*(V144C)	This work
pTdB-oL$_{I378C}$oM$_{P145C}$	*outL*(I378C) and *outM*(P145C)	This work
pTdB-oL$_{G381C}$oM$_{L122C}$	*outL*(G381C) and *outM*(L122C)	This work
pTdB-oL$_{G381C}$oM$_{A126C}$	*outL*(G381C) and *outM*(A126C)	This work
pTdB-oL$_{G381C}$oM$_{L139C}$	*outL*(G381C) and *outM*(L139C)	This work
pTdB-oL$_{G381C}$oM$_{L122C/L139C}$	*outL*(G381C) and *outM*(L122C/L139)	This work
pTdB-oL$_{G381C}$oM$_{V144C}$	*outL*(G381C) and *outM*(V144C)	This work
pTdB-oL$_{G381C}$oM$_{P145C}$	*outL*(G381C) and *outM*(P145C)	This work
pTdB-oL$_{G381C}$oM$_{L122C/P145C}$	*outL*(G381C) and *outM*(L122/P145C)	This work
pTdB-oL$_{G381C}$oM$_{A126C/P145C}$	*outL*(G381C) and *outM*(A126/P145C)	This work
