# Supplementary material for: Dynamic Interplay between the Periplasmic and Transmembrane Domains of GspL and GspM in the Type II Secretion System
Source: PLoS One. 2013 Nov 1;8(11):e79562. doi: 10.1371/journal.pone.0079562 (PMC3815138; doi:10.1371/journal.pone.0079562)
Supplement: Table S3 — Primers employed in the study. (PDF) [file pone.0079562.s007.pdf]

Table S3. Primers employed in the study

Primer	Nucleotide sequence (5'–3')[b]	Generated mutation
OuMC22I [a]	cgaatgatgacgctggcg***at***cggcggtctggtactgttg	OutMC22I
OuMC29V [a]	ggcggtctggtactgttg***gt***cctgctgtattatctgtgc	OutMC29V
OuMC35I [a]	gcctgctgtattatctg***at***ctgggcaccgtggcagg	OutMC35I
OuMY33C/C35I[a]	ctgttgtgcctgctgtatt***g***tctg***at***ctgggcaccgtgg	OutMY33C/C35I
OuML34C/C35I[a]	gttgtgcctgctgtattat***tgcat***ctgggcaccgtggcag	OutML34C/C35I
OuML122C [a]	cgtagcgatttcaacaacctg**tgc**cactggctggctgaactgg	OutM L122C
OuMA126C [a]	caacctgctgcactggctg**tg**tgaactggagcagaaaaacg	OutM A126C
OuML122C/ A126C [a]	caacctg**tg**ccactggctg**tg**tgaactggagcagaaaaacg	OutM L122C/A126C
OuML139C [a]	cggcgtgatcacccaggggt**gc**gatgtcacggcggttcccaacag	OutM L139C
OuMV141C [a]	cgtgatcacccaggggttggat**tg**cacggcggttcccaacagcgc	OutM V141C
OuMV144C [a]	caggggttggatgtcacggcg**tg**tcccaacagcgccggcatggtg	OutM V144C
OuMP145C [a]	gggttggatgtcacggcggtt**tg**caacagcgccggcatggtggag	OutM P145C
OuLI378C [a]	gcaggctcaggcatatttccag**tg**cccgccgggtgaaatgaagcag	OutL I378C
OuLP380C [a]	ctcaggcatatttccagatcccg**tgc**ggtgaaatgaagcaggaaaaagac	OutL P380C
OuLG381C [a]	gcatatttccagatcccgccg**t**gtgaaatgaagcaggaaaaag	OutL G381C
OuMXba	***gcgctcta***gaattgcgtcgccgctgg	*Xba*I, aa 2 of OutM
OuMER	***gaat***tcgcatcgattacagc	*Eco*RI, after *outM*
OuMHis	***catatgcaccatcaccatcaccat***aatgaattgcgtcgc	*Nde*I and 6His before *outM*
OuMNde5'	***ccggcatat***ggcaccgtggcaggagcg	*Nde*I before outM_{per}
OuMBH2 [a]	gctgtattatctgtgctggg***at***ccgtggcaggagcgggcg	*Bam*HI, aa 36 and 37 of OutM
OuMXba-sh [a]	gcacggcggcagatcgccgg***t***c***ta***gacatcagcctgacggtgttg	*Xba*I, aa 80 of OutM
OuMPst-sh [a]	gcaaagcgcggcccgct***g***c***a***gcatcaccgtgctgcgtatg	*Pst*I, aa 95 and 96 of OutM
OuMEcl136 [a]	gggcaccgtggcaggagc***tc***gcgcgacagtggcagatgac	*Ecl*136II, aa 42 of OutM
OuLHpa [a]	gtacgcgccggtgaaaccctg***tt***aacaggcggtgttgccgtggc	*Hpa*I, aa 241 and 242 of OutL
OuLNhe [a]	ggctgctgctggtgct***a***gcagagtctgtctggacccattac	*Nhe*I, aa 266 of OutL
OuLHis	***catatgcaccatcaccatcaccat***aacagggccgacaacg	*Nde*I and 6His before *outL*
OuLER	***ggga***attcatggctgactcctcag	*Eco*RI after *outL*
RCOuLNdRI	***gtcagtcacgatgaattcat at***gtgactcctcagaattaactg	*Nde*I and *Eco*RI after *outL*

[a] For each primers used in site directed mutagenesis, another primer with reverse complementary sequence was used (not shown).

[b] Mutated or introduced bases are in bold italic. Introduced restriction nuclease sites are underlined.
